# A joint Bayesian spatiotemporal risk prediction model of COVID-19 incidence, IC admission, and death with application to Sweden

**DOI:** 10.1007/s00168-022-01191-1

**Published:** 2022-11-28

**Authors:** I Gede Nyoman Mindra Jaya, Henk Folmer, Johan Lundberg

**Affiliations:** 1 Faculty of Spatial Sciences, University of Groningen, Groningen, The Netherlands; 2 Statistics Department, Padjadjaran University, Bandung, Indonesia; 3 Department of Economics and Centre for Regional Science (CERUM), Umeå University, 901 87 Umeå, Sweden

**Keywords:** C11, C35, I18

## Abstract

The three closely related COVID-19 outcomes of incidence, intensive care (IC) admission and death, are commonly modelled separately leading to biased estimation of the parameters and relatively poor forecasts. This paper presents a joint spatiotemporal model of the three outcomes based on weekly data that is used for risk prediction and identification of hotspots. The paper applies a pure spatiotemporal model consisting of structured and unstructured spatial and temporal effects and their interaction capturing the effects of the unobserved covariates. The pure spatiotemporal model limits the data requirements to the three outcomes and the population at risk per spatiotemporal unit. The empirical study for the 21 Swedish regions for the period 1 January 2020–4 May 2021 confirms that the joint model predictions outperform the separate model predictions. The fifteen-week-ahead spatiotemporal forecasts (5 May–11 August 2021) show a significant decline in the relative risk of COVID-19 incidence, IC admission, death and number of hotspots.

**Supplementary Information:**

The online version contains supplementary material available at 10.1007/s00168-022-01191-1.

## Introduction

Most people who have become ill with COVID-19 can recover at home. However, infected patients with severe symptoms need hospital care (Varghese et al. 2020). Clinical treatments include the administering of antiviral drugs (e.g. hydroxychloroquine or lopinavir/ritonavir) or the handling of complications including advanced organ support (e.g. supplemental oxygen and ventilatory support) (WHO 2020). A substantial proportion of the COVID-19 infected individuals deceases (Elezkurtaj et al. 2021).

Most of the literature on COVID-19 has focused on one of its outcomes separately, i.e. the number of incidences, or the number of IC admissions or the number of deaths. These outcomes are the three most important factors for the authorities to control as well as pieces of information for the public at large (Sahu and Böhning 2021). However, several studies on COVID-19 have suggested that the incidence, IC admission, and mortality curves parallel each other, although with lags and with unknown proportionality (IHME 2021; Ma et al. 2020; Onder et al. 2020). Nevertheless, so far, most studies have not taken into account the interplay between the outcomes by means of multivariate models (Congdon 2021). The aim of this paper is to (partly) fill this gap in that it jointly predicts and maps the three outcomes.

The joint prediction of the three outcomes taking into account their interplay has major advantages compared with separate modeling and prediction (Congdon 2021; Martins and Andreozzi 2016; Sahu and Böhning 2021). First, each equation in the joint model is affected by common socioeconomic and environmental factors, although usually with different impacts on the outcomes. In addition, there is a natural chronological order between the outcomes. Considering the simultaneity of the outcomes contributes to a more comprehensive understanding and prediction of the ongoing disease dynamics. Second, the three outcomes complement each other. For instance, the number of incidences is commonly associated with measurement error^1^ while the other two outcomes tend to be more accurately reported. By accounting for the associations between the outcomes, joint modeling reduces biased estimation of the parameters of the constituting separate equations, thus improving forecasting and mapping of each outcome (Chu 2021; Newalla et al. 2020; Scobie et al. 2021). Third, joint analysis is important from a policy and planning point of view. When the number of incidences increases, the numbers of hospitalizations and IC admissions and deaths follow suit, although with spatial and temporal lags with unknown proportionality (Borchering et al. 2021). Insight into the dependencies between the outcomes is basic information for the health authorities, hospitals and funeral enterprises to adequately manage their facilities and resources. The number of incidences is needed for the health autorities to issue warnings and implement behavioural restictions, for instance, distancing or lockdowns.^2^ Hospitals need predictions of the numbers of incidences as input for their predictions of hospitalizations, notably IC admissions, because COVID-19 patients require additional resources while the capacity is not easily adjusted in the short and medium term to meet higher demand. In a similar vein, funeral enterprises need predictions on the number of incidences, hospitalizations and IC admissions as inputs for their predictions of the number of deaths.

To capture similar patterns in the three disease outcomes, models with shared effects accounting for the interdependencies between the disease outcomes caused by common factors are required (Downing et al. 2008; Gomez-Rubio et al. 2019; Mahaki et al. 2018; Vicente et al. 2020; Serhiyenko et al. 2016). Deviations from the shared patterns due to outcome-specific factors are captured by specific effects (Knorr-Held and Best 2001).

The spread of COVID-19 varies both in time and space (Briz-Redón and Serrano-Aroca, 2020; Liu et al. 2020) making spatiotemporal modelling and mapping a necessity for adequate policy responses (Brett et al. 2018; Southall et al. 2020; Jaya and Folmer 2021a). The numbers of incidences, hospitalizations, IC admissions, and deaths per spatiotemporal unit are monitored on a daily or weekly basis in most countries. However, this does not apply to the main risk factors, notably demographic characteristics (Arani et al. 2021; Mutair et al. 2021), human behaviour (Chan et al. 2020; Nature 2020), socioeconomic conditions (Aleman et al. 2020; Cerqua and Letta 2022; Hawkins et al. 2020; Karmakar et al. 2021; Naylor-Wardle et al. 2021) and environmental variables (Azuma et al. 2020; Eslami and Jalili 2020; Lim et al. 2021; Poirier et al. 2020). The risk factors are commonly monitored for much larger time intervals than the three COVID-19 outcomes. However, for prediction purposes observations on the risk factors are *not* needed as their impacts can be inferred from the numbers of the COVID-19 outcomes, as reflected by their spatiotemporal trends and patterns which can be captured by hierarchical random effects models (Jaya and Folmer 2020, 2021a; Lopez-Qulez and Munoz 2009; Balamchi 2021; Choo and Walker 2008). The type of model based on spatiotemporal trends and patterns but without the risk factors is denoted as the pure model (Jaya and Folmer 2020; Lopez-Quılez and Munoz 2009).^3^ Particularly, if spatial and temporal autocorrelation and their interaction account for the majority of the space–time variation of the COVID-19 outcomes, one cannot expect a significant improvement in forecasting performance from an extension of the pure model by including covariates in the model (Lawson and Lee 2017; Lesaffre and Lawson 2012; Jaya and Folmer 2022a, under review; Turner and Witt 2001).^4^ Even more so, latent correlation between the random effects and the covariates frequently result in erroneous interpretations of the coefficients of the risk factors and deterioration of the prediction performance of the extended model.^5^ This applies especially to count data, as in the case of the COVID-19 outcomes (Jaya and Folmer 2022a, under review). On the other hand, if the research goal is to explicitly estimate the effects of the risk factors, then the covariates should be included in the model while the random effects capture residual spatiotemporal autocorrelation or heterogeneity. The model is denoted as the causal model (Jaya and Folmer 2022a, under review).^6^ Both the pure model and the causal model can be applied to identify spatiotemporal high-risk clusters (hotspots) (Jaya and Folmer 2020, 2021a, b; Lawson and Lee 2017; Lesaffre and Lawson 2012).

A spatiotemporal pure model consists of structured and unstructured spatial and temporal random effects and their interaction. The structured spatial and temporal random effects capture spatial and temporal correlation, respectively, the unstructured spatial and temporal random effects capture spatial and temporal heterogeneity, respectively. The four kinds of interaction between the spatial and temporal random effects (structured spatial effect × structured or unstructured temporal effect; unstructured spatial effect × structured or unstructured temporal effect) are captured by the interaction term (Knorr-Held 2000).

Pure spatiotemporal disease models can be adequately estimated using Bayesian statistics. Particularly, the Bayesian framework provides a convenient setting for hierarchical random effects models and forecasting. See amongst other Jaya and Folmer (2020, 2021b) and the references therein for details.

The objective of this paper is to jointly predict the relative risk of COVID-19 incidence, IC admission and death for Sweden based on regional weekly observations for the period 1 January 2020 – 4 May 2021. Because accurate data on hospitalization was not available, the prediction of the risk of the second outcome is restricted to IC admission. The use of regional data is motivated by policy considerations as the Swedish health care system is organized at the regional level. Joint out-of-sample spatiotemporal predictions will be compared to single equation predictions. In addition, we generate forecasts for the out-of sample period 5 May 2020 – 11 August 2021.

The paper is organized as follows. Section 2 discusses the joint spatiotemporal model, its Bayesian estimation and the prediction framework. Section 3 presents the data, descriptive statistics and the estimation results while Sect. 4 contains the predictions, including the exceedance probabilities and hotspots. Section 5 concludes.

## The joint Bayesian spatiotemporal COVID-19 risk model

Let ${y}_{it}$, ${o}_{it}$, and ${z}_{it}$ denote the numbers of confirmed COVID-19 incidences, IC admissions and deaths in region $i$ at time $t$, respectively, for $i = 1,\dots ,n$ and $t = 1,\dots , T$. We model ${y}_{it}$ marginally, ${o}_{it}$ conditionally on ${y}_{it}$ and *q* of its time lags and ${z}_{it}$ conditionally on ${y}_{it}$ and $r$ of its time lags.

We assume that the numbers of COVID-19 incidences, IC admissions, and deaths in region *i* in period *t*, follow Poisson distributions or, in the case of overdispersion, when the data contain large numbers of zeros, negative binomial (NB) distributions (Berk and MacDonald 2008; Payne et al. 2017), or zero-inflated Poisson (ZIP) distributions (Lewsey and Thomson 2004), or zero-inflated negative binomial (ZINB) distributions (Agarwal et al. 2002). For now, we assume that ${y}_{it}$, ${o}_{it}$, and ${z}_{it}$ follow Poisson distributions with means and variances equal to $\lambda_{it}^{y} = E_{it}^{y} \theta_{it}^{y}$, $\lambda_{it}^{o} = E_{it}^{o} \theta_{it}^{o}$, and $\lambda_{it}^{z} = E_{it}^{z} \theta_{it}^{z}$, respectively. Hence:

1a $$y_{it} \sim {\text{Poisson}}\left( {E_{it}^{y} \theta_{it}^{y} } \right){ }$$

1b $$o_{it} |y_{ik} \sim {\text{Poisson}}\left( {E_{it}^{o} \theta_{it}^{o} } \right)$$

1c $$z_{it} |y_{il} \sim {\text{Poisson}}\left( {E_{it}^{z} \theta_{it}^{z} } \right)$$

where $E_{it}^{h}$ and $\theta_{it}^{h}$ are the expected count and the relative risk of COVID-19 outcome $h$, respectively. (To avoid repetition, we use $h$ to collectively refer to the three outcomes. Hence, unless stated otherwise, $h= y,o,z$). The expected number of confirmed COVID-19 outcomes *h* is defined as:

2 $$E_{it}^{h} = N_{it}^{h} p^{h}$$

with $N_{{{\text{it}}}}^{h}$ denoting the number of persons at risk for outcome *h* and ${p}^{h}$ the constant probability of outcome *h* across all regions, respectively (Last 2001). ${N}_{\mathrm{it}}^{h}$ may be the entire population or a subset of the population, depending on susceptibility (Sellon and Long 2014).^7^ According to the WHO (2021), the entire population is susceptible to COVID-19 infection, IC admission, and death as a result of the coronavirus infection. Hence, we take the constant probability $p^{h}$ as (Abente et al. 2018; Jaya and Folmer 2020):

3 $$p^{h} = \frac{{\mathop \sum \nolimits_{i = 1}^{n} \mathop \sum \nolimits_{t = 1}^{T} h_{it} }}{{\mathop \sum \nolimits_{i = 1}^{n} \mathop \sum \nolimits_{t = 1}^{T} N_{it} }}{ }$$

A common definition of the relative risk is the standardized event ratio (${\text{SER}}^{h}$) defined as the ratio of the number of a confirmed cases $h$ and the corresponding expected count (Yin et al. 2014):

4 $${\text{SER}}_{{{\text{it}}}}^{h} = \frac{{h_{{{\text{it}}}} }}{{E_{{{\text{it}}}}^{h} }}$$

The standardized event ratio can be unreliable for sparse count data. Particularly, a spatiotemporal unit with a small number of observed outcomes and a small population at risk may be incorrectly classified as a high-risk unit (Jaya and Folmer 2017, 2020; Yin et al. 2014). To improve the relative risk estimate, we reduce spatiotemporal variation via smoothing by introducing spatiotemporal dependence and heterogeneity into the Poisson regression model, i.e. by borrowing strength across spatiotemporal units (Yin et al. 2014). Taking the logarithmic link function of the average number of events $\lambda_{{{\text{it}}}}^{h}$ yields:

5 $$\log \left( {\lambda_{it}^{h} } \right) = \log \left( {E_{it}^{h} } \right) + \log \left( {\theta_{it}^{h} } \right)$$

with $E_{it}^{h}$ the offset which is taken to have a regression coefficient fixed at 1. Using the offset as the denominator of the rate, the log relative risk reads (Blangiardo and Cameletti 2015):

6 $$\log \left( {\theta_{it}^{h} } \right) = \log \left( {\frac{{\lambda_{it}^{h} }}{{E_{it}^{h} }}} \right)$$

Accounting for spatiotemporal dependence and heterogeneity, the log relative risk for each outcome in Eq. (1) takes the form of a generalized linear mixed model as follows:

7a $$\eta_{it}^{y} = \log \theta_{it}^{y} = \alpha^{y} + \omega_{i}^{y} + \upsilon_{i}^{y} + \phi_{t}^{y} + \varsigma_{t}^{y} + \delta_{it}^{y}$$

7b $$\eta_{it}^{o} = \log \theta_{it}^{o} = \alpha^{o} + \mathop \sum \limits_{k = 0}^{q} \gamma_{1,\,\,\,\,k + 1} \log \left( {\frac{{y_{i,t - k} }}{{E_{i,\,\,\,\,t - k}^{y} }}} \right) + \omega_{i}^{o} + \upsilon_{i}^{o} + \phi_{t}^{o} + \varsigma_{t}^{o} + \delta_{it}^{o}$$

7c $$\eta_{it}^{z} = \log \theta_{it}^{z} = \alpha^{z} + \mathop \sum \limits_{l = 0}^{r} \gamma_{2,\,\,\,\,l + 1} \log \left( {\frac{{y_{i,\,\,\,\,t - l} }}{{E_{i,t - l}^{y} }}} \right) + \omega_{i}^{z} + \upsilon_{i}^{z} + \phi_{t}^{z} + \varsigma_{t}^{z} + \delta_{it}^{z}$$

where ${\alpha }^{h}$ is the intercept, ${\omega }_{i}^{h} \mathrm{and} {\upsilon }_{i}^{h}$ are the structured and unstructured spatial random effects, respectively,${\phi }_{t}^{h} \mathrm{and} {\varsigma }_{t}^{h}$ the structured and unstructured temporal random effects, respectively, and ${\delta }_{it}^{h}$ the interaction effect capturing the interaction between a pair of structured or unstructured spatial and temporal random effects (Knorr-Held 2000). The random effects capture the effects of the unobserved risk factors (Jaya and Folmer 2020; Kazembe 2007) while the lagged variables $\mathrm{log}\left(\frac{{y}_{i,t-k}}{{E}_{i,t-k}^{y}}\right) \mathrm{and} \mathrm{log}\left(\frac{{y}_{i,t-l}}{{E}_{i,t-l}^{y}}\right)$ account for the time lags of incidence risk for IC admission $({o}_{it}$) and death (${z}_{it}$), respectively.^8^

Models (7a)–(7c) can be combined into a single model by stacking the outcomes as $\varvec{g} = \left( {\varvec{y}^{\prime } ,\varvec{o}^{\prime } ,\varvec{z}^{\prime } } \right)^{\prime } = \left( {g_{{11}}^{y} , \ldots ,g_{{nT}}^{y} ,g_{{11}}^{o} , \ldots ,g_{{nT}}^{o} ,g_{{11}}^{z} \ldots ,g_{{nT}}^{z} } \right)^{\prime }$, the population at risk as $\varvec{E} = \left( {\varvec{E}^{{\varvec{y}^{\prime } }} ,\varvec{E}^{{\varvec{o}^{\prime } }} ,\varvec{E}^{{\varvec{z}^{\prime } }} } \right)^{\prime } = \left( {E_{{11}}^{y} , \ldots ,E_{{nT}}^{y} ,E_{{11}}^{o} , \ldots ,E_{{nT}}^{o} ,E_{{11}}^{z} , \ldots ,E_{{nT}}^{z} } \right)^{\prime }$ and the log relative risk as ${\varvec{\eta}} = \left( {\log \left( {\theta_{11}^{y} } \right), \ldots ,\log \left( {\theta_{nT}^{y} } \right),\log \left( {\theta_{11}^{o} } \right), \ldots ,\log \left( {\theta_{nT}^{o} } \right),\log \left( {\theta_{11}^{z} } \right), \ldots ,\log \left( {\theta_{nT}^{z} } \right)} \right)^{\prime}.$ According to Gomez-Rubio et al. (2019) and Knorr-Held and Best (2001), many disease outcomes share common risk factors and thus have similar spatiotemporal patterns of variation. To capture similar spatiotemporal patterns (clusters) in the outcomes, structured spatial variation and structured temporal variation are decomposed into specific and shared effects (Gomez-Rubio et al. 2019; Knorr-Held and Best 2001). Specifically, the structured spatial effect is defined as $\omega_{i}^{h} = \ddot{\omega }_{i}^{h} + \varphi^{h} \tilde{\omega }_{i}$ and the structured temporal effect as $\phi_{i}^{h} = \ddot{\phi }_{i}^{h} + \varrho^{h} \tilde{\phi }_{i}$ with $\ddot{\omega }_{i}^{h}$ and $\ddot{\phi }_{i}^{h}$ denoting the structured spatial and structured temporal specific effect, respectively, and $\tilde{\omega }_{i}$ and ${\widetilde{\phi }}_{t}$ the structured spatial and structured temporal shared effect, respectively, with coefficients ${\varphi }^{h}$ and${\varrho }^{h}$, respectively, representing their impacts on outcome $h,$ respectively. Including the structured spatial and structured temporal specific ($\ddot{\omega }_{i}^{h}$ and $\ddot{\phi }_{t}^{h}$) and shared effects ($\tilde{\omega }_{i}$ and $\tilde{\phi }_{t}$) with weights $\varphi^{h}$ and $\varrho^{h}$ in $\eta_{it}^{h}$, Eqs. (7a–c) can be jointly written as: 9

8 $$\begin{aligned} \eta _{{it}}^{h} = & \alpha ^{h} + I_{o} \left( {\mathop \sum \limits_{{k = 0}}^{q} \gamma _{{1,k + 1}} \log \left( {\frac{{y_{{i,t - k}} }}{{E_{{i,t - k}}^{y} }}} \right)} \right) + ~I_{z} \left( {\mathop \sum \limits_{{l = 0}}^{r} \gamma _{{2,l + 1}} \log \left( {\frac{{y_{{i,t - l}} }}{{E_{{i,t - l}}^{y} }}} \right)} \right) \\ ~~~~~~~~~~~~~~~~~~~~~~ & + \;~\left( {~\ddot{\omega }_{i}^{h} + \varphi ^{h} \tilde{\omega }_{i} } \right) + \upsilon _{i}^{h} + \left( {\ddot{\phi }_{t}^{h} + \rangle ^{h} \tilde{\phi }_{t} } \right) + \varsigma _{t}^{h} + \delta _{{it}}^{h} \\ \end{aligned}$$

with $\left\{ {\alpha^{h} , \upsilon_{i}^{h} ,\varsigma_{t}^{h} ,\delta_{it}^{h} } \right\}$ defined in Eqs. (7a–7c), $I_{o}$ and $I_{z}$ the indicator functions for IC admission and death, respectively. We take model (8) as a hierarchical Bayesian latent Gaussian model (LGM) consisting of fixed and random effects (Rue et al. 2017). The coefficients of the fixed effects ($\gamma_{1,\,\,\,\,k + 1}$ and $\gamma_{2,\,\,\,\,l + 1} )$ present the impacts of the time lags of incidence on the IC admission and death, respectively. The random effects (i.e., $\ddot{\omega }_{i}^{h} ,\tilde{\omega }_{i} ,\upsilon_{i}^{h} ,\ddot{\phi }_{t}^{h} ,\tilde{\phi }_{t} ,\varsigma_{t}^{h} , {\text{and }}\delta_{it}^{h}$) account for the structured spatial specific and shared effects, unstructured spatial effects, structured temporal specific and shared effects, unstructured temporal effects, and spatiotemporal interaction effect, respectively.

For estimation of model (8), we consider (i) the likelihood of the data, (ii) the prior distributions for the parameters which are assumed to follow Gaussian distributions (iii) the hyperprior distributions for the hyperparameters which are also assumed to follow Gaussian distributions. Regarding (i), the observations of outcome $h$ in region $i$ at time $t$, ${g}_{it}^{h}$, are conditionally independent^10^ of the observations of the other outcomes given the linear predictors $\eta_{it}^{h}$. Hence, from Eqs. (1a–1c) and (8), we have:

9 $$g_{it}^{h} |\eta_{it}^{h} \sim iid\,\,\,\,{\text{Poisson}}\left( {E_{it}^{h} \exp \left( {\eta_{it}^{h} } \right)} \right)$$

with ${\eta }_{it}^{h}$ defined in Eq. (8).

Regarding (ii), we assign vague Gaussian prior distributions to the parameters $\alpha^{y}$, $\alpha^{o}$, $\alpha^{z}$, $\gamma_{1,1} , \ldots ,\gamma_{1,q + 1}$, $\gamma_{2,1} , \ldots ,\gamma_{2,r + 1}$, $\varphi^{y} ,\varphi^{o} ,\varphi^{z} ,$ $\varrho^{y}$, $\varrho^{o}$ and $\varrho^{z}$ (Jaya and Folmer 2021a; Martinez-Beneito and Botella-Rocamora 2019). For the structured spatial specific effects the intrinsic conditional autoregressive model (iCAR) (Besag et al. 1991) and the more general Leroux CAR (LCAR)^11^ model (Leroux et al. 2000) are common prior distributions.^12^ The LCAR is an extension of the iCAR and is applicable to a wider variety of spatial correlation scenarios (Lee 2011). The iCAR prior for $\ddot{\omega }_{i}^{h}$ is (Besag et al. 1991):

10 $$\ddot{\omega }_{i}^{h} |\ddot{\omega }_{ - i}^{h} ,\sigma_{{\ddot{\omega }^{h} }}^{2} ,{\mathbf{W}}\sim {{\mathcal{N}}}\left( {\frac{{\mathop \sum \nolimits_{j = 1}^{n} w_{ij} \ddot{\omega }_{j}^{h} }}{{\mathop \sum \nolimits_{i = 1}^{n} w_{ij} }},\frac{{\sigma_{{\ddot{\omega }^{h} }}^{2} }}{{\mathop \sum \nolimits_{i = 1}^{n} w_{ij} }}} \right)$$

where $w_{ij}$ is an element of the spatial weights matrix **W** defined as:

$$w_{ij} = \left\{ {\begin{array}{*{20}c} {1\,\,\,\,{\text{if}}\,\,\,\,i\,\,\,\,{\text{and }}j\,\,\,\,{\text{are adjacent neighbors}}} \\ {0 \,\,\,\,{\text{otherwise}} } \\ \end{array} } \right.$$

and $\sigma_{{\ddot{\omega }^{h} }}^{2}$ is the variance parameter of $\ddot{\omega }_{i}^{h}$. The LCAR prior reads (Leroux et al. 2000):

11 $$\ddot{\omega }_{i}^{h} |\ddot{\omega }_{ - i}^{h} ,\sigma_{{\ddot{\omega }^{h} }}^{2} ,{\mathbf{W}}\sim {{\mathcal{N}}}\left( {\frac{{\rho^{h} \mathop \sum \nolimits_{j = 1}^{n} w_{ij} \ddot{\omega }_{j}^{h} }}{{\rho^{h} \mathop \sum \nolimits_{j = 1}^{n} w_{ij} + 1 - \rho^{h} }},\frac{{\sigma_{{\ddot{\omega }^{h} }}^{2} }}{{\left( {\rho^{h} \mathop \sum \nolimits_{j = 1}^{n} w_{ij} + 1 - \rho^{h} } \right)}}} \right)$$

where ${\rho }^{h}$ denotes the spatial autoregressive parameter. (The iCAR prior is the limiting case of the Leroux prior when ${\rho }^{h}$ equals 1). The structured spatial shared effect ${\widetilde{\omega }}_{i}$ is also assigned a CAR (iCAR or LCAR)) prior. The unstructured spatial effects ${v}_{i}$ are assigned exchangeable Normal priors (Osei and Stein 2019)^13^:

12 $$\upsilon_{i}^{h} |\sigma_{{\upsilon^{h} }}^{2} \sim {{\mathcal{N}}}\left( {0,\sigma_{{\upsilon^{h} }}^{2} } \right)$$

where $\sigma_{{\upsilon^{h} }}^{2}$ is the variance parameter of $\upsilon_{i}^{h}$. A common prior for the structured temporal specific effects $\ddot{\phi }_{t}^{h}$ is a random walk of order one (RW1) (Schrödle and Held 2011):

13 $$\ddot{\phi }_{t + 1}^{h} - \ddot{\phi }_{t}^{h} |\sigma_{{\ddot{\phi }^{h} }}^{2} \sim {{\mathcal{N}}}\left( {0,\sigma_{{\ddot{\phi }^{h} }}^{2} } \right){ }$$

with $\sigma_{{\ddot{\phi }^{h} }}^{2}$ the variance parameter of $\ddot{\phi }_{t}^{h}$. An alternative to the RW1 prior when the data has a pronounced linear trend is the random walk of order two (RW2). It reads as:

14 $$\ddot{\phi }_{t}^{h} - 2\ddot{\phi }_{t + 1}^{h} + \ddot{\phi }_{t + 2}^{h} |\sigma_{{\ddot{\phi }^{h} }}^{2} \sim {{\mathcal{N}}}\left( {0,\sigma_{{\ddot{\phi }}}^{2} } \right)$$

Note that both RW priors are cyclic accounting for irregular patterns of fluctuation in the data (Gómez-Rubio 2020; Riebler et al. 2011). The structured temporal shared effects $\tilde{\phi }_{t}$ are also assigned RW (i.e. RW1 or RW2) priors. For the unstructured temporal ${\varsigma }_{t}^{h}$ we assume exchangeable Normal priors (Schrödle and Held 2010):

15 $$\varsigma_{t}^{h} |\sigma_{{\varsigma^{h} }}^{2} \sim {{\mathcal{N}}}\left( {0,\sigma_{{\varsigma^{h} }}^{2} } \right)$$

where ${\sigma }_{{\varsigma }^{h}}^{2}$ is the variance parameter of ${\varsigma }_{t}^{h}$. For spatiotemporal interaction we choose the interaction structured spatial random effect × structured temporal random effect with corresponding priors. The interaction captures deviations from the shared spatial and temporal trend (Iddrisu et al. 2018; Knorr-Held 2000).

Regarding (iii), the spatial autoregressive hyperparameter $\rho^{h}$ of the LCAR prior and the variances $\sigma_{{\ddot{\omega }^{h} }}^{2} ,\sigma_{{\tilde{\omega }}}^{2} , \sigma_{{\upsilon^{h} }}^{2} ,\sigma_{{\ddot{\phi }^{h} }}^{2} ,\sigma_{{\tilde{\phi }}}^{2} ,\sigma_{{\varsigma^{h} }}^{2}$ and $\sigma_{{\delta^{h} }}^{2}$ require prior distributions, called hyperpriors. As hyperprior for $\rho^{h}$ in Eq. (11), we select $\log \left( {\rho^{h} /\left( {1 - } \right)} \right)\sim {{\mathcal{N}}}\left( {0,0.450} \right)$ (Blangiardo and Cameletti 2015). (The transformation is used to ensure that $\rho^{h}$ takes values between 0 and 1 (Bivand et al. 2015; Martinez-Beneito and Botella-Rocamora 2019)). The weakly informative half-Cauchy distribution with scale parameter 25 is assigned to the square roots of the variances $\{ \sigma_{{\ddot{\omega }^{h} }}^{2} ,\sigma_{{\tilde{\omega }}}^{2} , \sigma_{{\upsilon^{h} }}^{2} ,\sigma_{{\ddot{\phi }^{h} }}^{2} ,\sigma_{{\tilde{\phi }}}^{2} ,\sigma_{{\varsigma^{h} }}^{2}$, $\sigma_{{\delta^{h} }}^{2} \}$ (Gelman 2006).

$$\begin{aligned} {\text{Let}}\,\,\,\,\Omega & = (\eta _{{11}}^{y} , \ldots ,\eta _{{nT}}^{y} ,\eta _{{11}}^{o} , \ldots ,\eta _{{nT}}^{o} ,\eta _{{11}}^{z} , \ldots ,\eta _{{nT}}^{z} ,\alpha ^{y} ,\alpha ^{z} ,\gamma _{{1,1}} , \ldots ,\gamma _{{1,q + 1}} ,\gamma _{{1,1}} , \ldots ,\gamma _{{1,r + 1}} , \\ \ddot{\omega }_{1}^{o} , \ldots ,\ddot{\omega }_{n}^{o} ,\ddot{\omega }_{1}^{z} , \ldots ,\ddot{\omega }_{n}^{z} ,\tilde{\omega }_{1} , \ldots ,\tilde{\omega }_{N} ,\upsilon _{1}^{y} , \ldots ,\upsilon _{n}^{y} ,\upsilon _{1}^{o} , \ldots ,\upsilon _{n}^{o} ,\upsilon _{1}^{z} , \ldots ,\upsilon _{n}^{z} ,\ddot{\phi }_{1}^{y} , \ldots ,\ddot{\phi }_{T}^{y} ,\ddot{\phi }_{1}^{o} , \ldots ,\ddot{\phi }_{T}^{o} ,\ddot{\phi }_{1}^{z} , \ldots ,\ddot{\phi }_{T}^{z} ,\tilde{\phi }_{1} , \ldots ,\tilde{\phi }_{T} , \\ \varsigma _{1}^{y} , \ldots ,\varsigma _{T}^{y} ,\varsigma _{1}^{o} , \ldots ,\varsigma _{T}^{o} ,\varsigma _{1}^{z} , \ldots ,\varsigma _{T}^{z} ,\delta _{{11}}^{y} , \ldots ,\delta _{{nT}}^{y} ,\delta _{{11}}^{o} , \ldots ,\delta _{{nT}}^{o} ,\delta _{{11}}^{z} , \ldots ,\delta _{{nT}}^{z} ,\varphi ^{y} ,\varphi ^{o} ,\varphi ^{z} ,\rangle ^{y} ,\rangle ^{o} ,\rangle ^{z} )^{\prime } \\ \Psi & = \sigma _{{\ddot{\omega }^{y} }}^{2} ,\sigma _{{\ddot{\omega }^{o} }}^{2} ,\sigma _{{\ddot{\omega }^{z} }}^{2} ,\sigma _{{\tilde{\omega }}}^{2} ,\sigma _{{\upsilon ^{y} }}^{2} ,\sigma _{{\upsilon ^{o} }}^{2} ,\sigma _{{\upsilon ^{z} }}^{2} ,\sigma _{{\ddot{\phi }^{y} }}^{2} ,\sigma _{{\ddot{\phi }^{o} }}^{2} ,\sigma _{{\ddot{\phi }^{z} }}^{2} ,\sigma _{{\tilde{\phi }}}^{2} ,\sigma _{{\varsigma ^{y} }}^{2} ,\sigma _{{\varsigma ^{o} }}^{2} ,\sigma _{{\varsigma ^{z} }}^{2} ,\sigma _{{\delta ^{y} }}^{2} ,\sigma _{{\delta ^{o} }}^{2} ,\sigma _{{\delta ^{z} }}^{2} ,\rho ^{y} ,\rho ^{o} ,\rho ^{z} )^{\prime } \\ \end{aligned}$$ be the vectors of the parameters and hyperparameters, respectively. The joint posterior distribution of the joint Bayesian spatiotemporal model in Eq. (8) is then given by:

16 $$p\left( {{{\varvec{\Omega}}},{{\varvec{\Psi}}}|{\mathbf{g}}} \right) = \frac{{p\left( {{\mathbf{g}}|{{\varvec{\Omega}}},{{\varvec{\Psi}}}} \right)p\left( {{{\varvec{\Omega}}}|{{\varvec{\Psi}}}} \right)p\left( {{\varvec{\Psi}}} \right)}}{{p\left( {{\mathbf{g}}|{{\varvec{\Psi}}}} \right)}} \propto p\left( {{\mathbf{g}}|{{\varvec{\Omega}}},{{\varvec{\Psi}}}} \right)p\left( {{{\varvec{\Omega}}}|{{\varvec{\Psi}}}} \right)p\left( {{\varvec{\Psi}}} \right)$$

where $p\left(\mathbf{g}|{\varvec{\Omega}},{\varvec{\Psi}}\right)$ denotes the conditionally independent likelihood function given the linear predictors and ${\varvec{\Omega}}\mathrm{and }{\varvec{\Psi}},$ $p\left({\varvec{\Omega}}|{\varvec{\Psi}}\right)$ is the prior distribution of ${\varvec{\Omega}}$ given ${\varvec{\Psi}},$ and $p({\varvec{\Psi}})$ is the hyperprior distributions $\mathrm{and} p(\mathbf{g}|{\varvec{\Psi}})$ the marginal likelihood. The latter is a normalizing constant and can be ignored as it does not include the parameter vector ${\varvec{\Omega}}$.

For the joint likelihood function $p\left(\mathbf{g}|{\varvec{\Omega}},{\varvec{\Psi}}\right)$, the vector of observed disease outcomes, ${\varvec{g}},$ is conditionally independent given ${\varvec{\Omega}}$ and ${\varvec{\Psi}}$ **(**see note 10**)**. Hence, the likelihood can be expressed as:

17 $$p\left( {{\mathbf{g}}|{{\varvec{\Omega}}},{{\varvec{\Psi}}}} \right) = \mathop \prod \limits_{l = 1}^{n} \mathop \prod \limits_{t = 1}^{T} p\left( {{\mathbf{g}}_{it} |{{\varvec{\Omega}}},{{\varvec{\Psi}}}} \right) = \mathop \prod \limits_{l = 1}^{n} \mathop \prod \limits_{t = 1}^{T} p\left( {{\text{y}}_{it} |{{\varvec{\Omega}}},{{\varvec{\Psi}}}} \right)p\left( {{\text{o}}_{it} |{{\varvec{\Omega}}},{{\varvec{\Psi}}}} \right)p\left( {{\text{z}}_{it} |{{\varvec{\Omega}}},{{\varvec{\Psi}}}} \right)$$

For the second component of the joint posterior distribution in Eq. (16), $p\left( {{{\varvec{\Omega}}}|{{\varvec{\Psi}}}} \right)$, we assume a multivariate Normal prior for ${{\varvec{\Omega}}}$ with mean $0$ and precision^14^ matrix **Q**(${{\varvec{\Psi}}}$). Hence:

18 $$p\left( {{{\varvec{\Omega}}}|{{\varvec{\Psi}}}} \right) \propto \left| {{\mathbf{Q}}\left( {{\varvec{\Psi}}} \right)} \right|^{\frac{1}{2}} \exp \left( { - \frac{1}{2}{\mathbf{\Omega^{\prime}Q}}\left( {{\varvec{\Psi}}} \right){{\varvec{\Omega}}}} \right),$$

where $\left| . \right|$ denotes the determinant. The latent Gaussian field ${{\varvec{\Omega}}}$ is taken as a Gaussian Markov random field (GMRF) (Rue and Leonhard-Held 2005; Sidén et al. 2018). Thus ${\mathbf{Q}}_{{uu^{\prime}}} \ne 0$ only if $u\prime$ = $u$ or ${u}^{^{\prime}}$ is an immediate neighbour of $u, \mathrm{for} u=1,..U\mathrm{ with }U$ the number of elements of${\varvec{\Omega}}$. Finally, the hyperparameters controlling the variability of the parameters are assumed to be independent of each other implying that the joint hyperprior $p\left({\varvec{\Psi}}\right)$ $=\prod_{s=1}^{S}p\left({\Psi }_{s}\right)$ with $S$ the number of elements of ${\varvec{\Psi}}$. Combining Eqs. (16–18) and the multiplicative joint hyperprior $p\left({\varvec{\Psi}}\right)$ gives:

19 $$\begin{gathered} p\left( {{{\varvec{\Omega}}},{{\varvec{\Psi}}}|{\mathbf{g}}} \right) \propto \mathop \prod \limits_{l = 1}^{n} \mathop \prod \limits_{t = 1}^{T} p\left( {{\text{y}}_{it} |{{\varvec{\Omega}}},{{\varvec{\Psi}}}} \right)p\left( {{\text{o}}_{it} |{{\varvec{\Omega}}},{{\varvec{\Psi}}}} \right)p\left( {{\text{z}}_{it} |{{\varvec{\Omega}}},{{\varvec{\Psi}}}} \right) \hfill \\ \times \left| {{\mathbf{Q}}\left( {{\varvec{\Psi}}} \right)} \right|^{\frac{1}{2}} \exp \left( { - \frac{1}{2}{\mathbf{\Omega^{\prime}Q}}\left( {{\varvec{\Psi}}} \right){{\varvec{\Omega}}}} \right) \times \mathop \prod \limits_{s = 1}^{S} p\left( {{\Psi }_{s} } \right) \hfill \\ \propto \exp \left( {\mathop \sum \limits_{i = t}^{n} \mathop \sum \limits_{t = 1}^{T} \log \left( {p\left( {{\text{y}}_{it} |{{\varvec{\Omega}}},{{\varvec{\Psi}}}} \right)p\left( {{\text{o}}_{it} |{{\varvec{\Omega}}},{{\varvec{\Psi}}}} \right)p\left( {{\text{z}}_{it} |{{\varvec{\Omega}}},{{\varvec{\Psi}}}} \right)} \right) - \frac{1}{2}{\mathbf{\Omega^{\prime}Q}}\left( {{\varvec{\Psi}}} \right){{\varvec{\Omega}}}} \right) \hfill \\ \times \left| {{\mathbf{Q}}\left( {{\varvec{\Psi}}} \right)} \right|^{\frac{1}{2}} \mathop \prod \limits_{s = 1}^{S} p\left( {{\Psi }_{s} } \right) \hfill \\ \end{gathered}$$

The posterior density in Eq. (19) can be calculated applying integrated nested Laplace approximation (INLA) using the R-INLA package (Rue et al. 2009; Jaya and Folmer 2020, 2021a).^15^ Instead of considering the full posterior distributions of ${\varvec{\Omega}}$ and ${\varvec{\Psi}}$, INLA proceeds on the basis of approximations to the marginal posterior distributions $p\left({\Omega }_{u}|\mathbf{g}\right)$ and $p\left({\Psi }_{s}|\mathbf{g}\right)$. The marginal posterior distribution of ${\Omega }_{u}$ is:

20 $$p\left( {{\Omega }_{u} |{\mathbf{g}}} \right) = \smallint p\left( {{\Omega }_{u} |{{\varvec{\Psi}}},{\mathbf{g}}} \right)p\left( {{{\varvec{\Psi}}}|{\mathbf{g}}} \right)d{{\varvec{\Psi}}}$$

and the marginal posterior distribution of ${\Psi }_{s}$ is:

21 $$p\left( {{\Psi }_{s} |{\mathbf{g}}} \right) = \smallint p\left( {{{\varvec{\Psi}}}|{\mathbf{g}}} \right)d{{\varvec{\Psi}}}_{{ - {\varvec{s}}}}$$

The following tasks must be performed to obtain Eqs. (20) and (21): (i) compute $p\left({\varvec{\Psi}}|\mathbf{g}\right)$ from which all the marginal posteriors $p\left({\Psi }_{s}|\mathbf{g}\right)$ can be calculated, and (ii) compute $p\left({\Omega }_{u}|{\varvec{\Psi}},\mathbf{g}\right)$ from which the marginal posteriors of $p\left({\Omega }_{u}|\mathbf{g}\right)$ are calculated. Hence, the posterior means, standard deviations, forecasts, and other statistics are obtained from their marginal posterior distributions $p\left({\Omega }_{u}|\mathbf{g}\right)$.

The R-INLA package also provides goodness-of-fit statistics, notably the deviance information criterion (DIC) (Spiegelhalter et al. 2002) and the Watanabe Akaike information criterion (WAIC) (Watanabe 2010). It also yields the marginal predictive likelihood (MPL) (Dey et al. 1997), mean absolute error (MAE), root mean square error (RMSE) (Pal 2017), and Pearson correlation coefficient (*r*) (Santa et al. 2019), which are appropriate statistics for prediction performance evaluation.

Forecasts of the relative risk of incidence, IC admission and death in a joint Bayesian setting are based on the posterior predictive distributions $p\left({\widehat{\theta }}_{it}^{h}|\mathbf{g}\right)$ (Wang et al. 2018). Forecasting in R-INLA can be handled by fitting a model with missing observations. Specifically, one combines observed values with missing or unavailable (NA) observations for the forecast periods (see amongst others Jaya and Folmer 2020, 2021b). For the prediction of a variable at $t=1$, the most recent information (at time $t=0$) and previous information ($t<0$) is used. For the prediction for $t>1$, say $t=s$, the information for the prediction at $t=1$ is used, plus the predictions at $t=1, 2,\dots ,s-1$ (recursive updating).

As mentioned in the Introduction, hotspots are of special interest from a policy point of view. They can be identified using the exceedance probability (Jaya and Folmer 2021a; Lawson 2010) which is defined as the probability that the estimated posterior mean of the relative risk of outcome $h$ in region $i$ at time $t$ is greater than a threshold value $c$, that is, $\mathrm{Pr}\left({\widehat{\theta }}_{it}^{h}>c|\mathbf{g}\right)$. It is calculated as:

22 $${\text{Pr}}\left( {\hat{\theta }_{{it}}^{h} {\text{ < }}c|{\mathbf{g}}} \right) = 1 - \int\limits_{{\hat{\theta }_{{it}}^{h} \le c}} {p\left( {\hat{\theta }_{{it}}^{h} |{\mathbf{g}}} \right)d\theta _{{it}}^{h} }$$

where $\int\limits_{{\hat{\theta }_{{it}}^{h} \le c}} {p(\hat{\theta }_{{it}}^{h} \left| \varvec{g} \right.){\text{d}}\hat{\theta }_{{it}}^{h} }$ is the cumulative probability of $\hat{\theta }_{it}^{h}$ given ${\mathbf{g}} = \left( {\varvec{y^{\prime}},\varvec{o^{\prime}},\varvec{z^{\prime}}} \right)^{\prime}$ and the threshold value $c.$ To identify spatiotemporal hotspot we also need the cut-off value of the exceedance probability, denoted $\gamma .$ Hence, a spatiotemporal unit is classified as a hotspot if ${\text{Pr}}\left( {\hat{\theta }_{it}^{h} > c|{\mathbf{g}}} \right)$ > $\gamma$. Common values for $c$ are $1$–3, for $\gamma$ $0.90$ and 0.95 (Lawson and Rotejanaprasert 2014). For further details, see Jaya and Folmer (2021a).

## The joint COVID-19 prediction model for the Swedish regions, 1 January 2020–4 May 2021

The empirical analysis is based on weekly COVID-19 data provided by the Public Health Agency of Sweden (PHAS), covering all 21 Swedish regions ($n=21$) from 1 January 2020 until 4 May 2021 ($T=70$). COVID-19 incidences, $\left(y\right)$, are the confirmed weekly numbers of new cases with COVID-19 infection according to the Swedish case definition (PHAS, 2021) and reported in accordance with the Infection Control Act of COVID-19. IC admissions, $\left(o\right),$ are the weekly numbers of new patients with a laboratory-confirmed COVID-19 infection admitted to intensive care. Deaths $\left(z\right)$ are the weekly numbers of deceased persons with a laboratory-confirmed COVID-19 diagnosis and reported as deceased in the database SmiNet. The weekly numbers and rates per 100,000 inhabitants of incidences, IC admissions, and deaths are presented in Fig. 5, Appendix 1. A summary of the data is presented in Table 1 .

**Table 1:** Accumulated numbers and rates of COVID-19 incidences, IC admissions and deaths per region from 1 January 2020 to 4 May 2021

Region	Number			Population at risk	Rate (per 100,000 inhabitants)		
	Incidences	IC admission	Death		Incidences	IC admission	Death
Blekinge	11,493	49	128	159,606	7,201	31	80
Dalarna	24,225	168	332	287,966	8,412	58	115
Gävleborg	33,335	231	546	287,382	11,600	80	190
Gotland	3,654	30	49	59,686	6,122	50	82
Halland	38,187	163	310	333,848	11,438	49	93
Jämtland/Härjedalen	11,075	63	120	130,810	8,466	48	92
Jönköping	39,574	315	545	363,599	10,884	87	150
Kalmar	21,107	99	229	245,446	8,599	40	93
Kronoberg	19,198	111	306	201,469	9,529	55	152
Norrbotten	21,148	179	254	250,093	8,456	72	102
Örebro	28,782	211	326	304,805	9,443	69	107
Östergötland	37,287	313	610	465,495	8,010	67	131
Skåne	142,571	581	1,687	1,377,827	10,348	42	122
Sörmland	23,139	330	445	297,540	7,777	111	150
Stockholm	233,738	2,029	4,225	2,377,081	9,833	85	178
Uppsala	37,056	375	520	383,713	9,657	98	136
Värmland	16,928	103	199	282,414	5,994	36	70
Västerbotten	21,139	143	178	271,736	7,779	53	66
Västernorrland	24,168	155	456	245,347	9,851	63	186
Västmanland	24,937	107	343	275,845	9,040	39	124
Västra Götaland	172,742	1,275	2,306	1,725,881	10,009	74	134
Global	985,483	7,030	14,114	10,327,589	9,542	68	137

Source: Public Health Agency of Sweden (PHAS) (https://www.folkhalsomyndigheten.se/the-public-health-agency-of-sweden/communicable-disease-control/covid-19/)

**Fig. 1:**
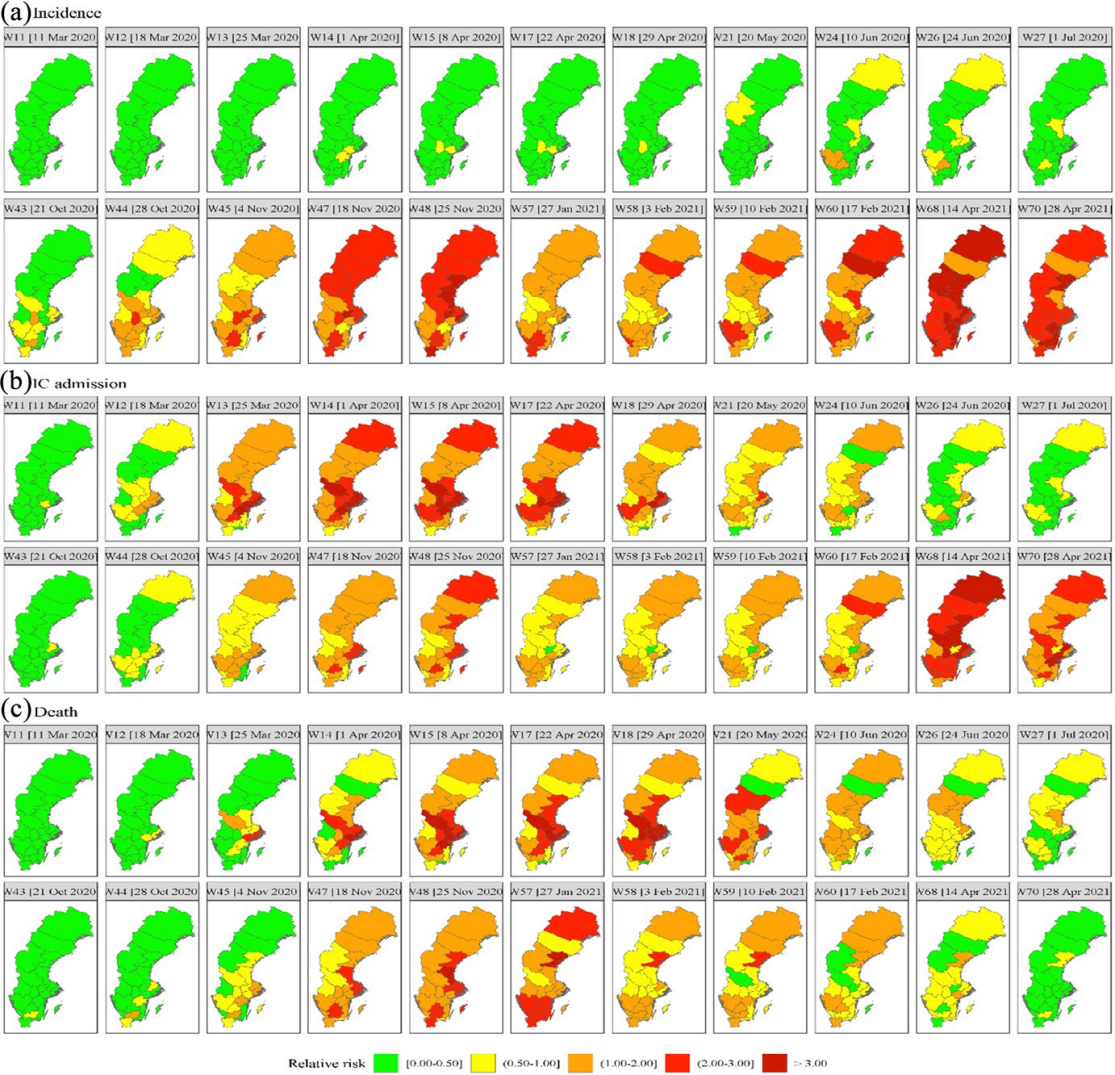
Posterior means of the relative risk for (a) incidence, (b) IC admission, and (c) Death for selected weeks for W11-15 (11 Mar 2020–8 Apr 2020), W17 (22 Apr 2020), W18 (29 Apr 2020), W21 (20 May 2020), W24 (10 Jun 2020), W26 (24 Jun 2020), W27 (1 Jul 2020), W43 (21 Oct 2020), W44 (28 Oct 2020), W45 (4 Nov 2020), W47 (18 Nov 2020), W48 (25 Nov 2020), W57 (27 Jan 2021), W58 (3 Feb 2021), W59 (10 Feb 2021), W60 (17 Feb 2021), W68 (14 Apr 2021), W70 (28 Apr 2021). W refers to the week and the date to the beginning of the week

**Fig. 2:**
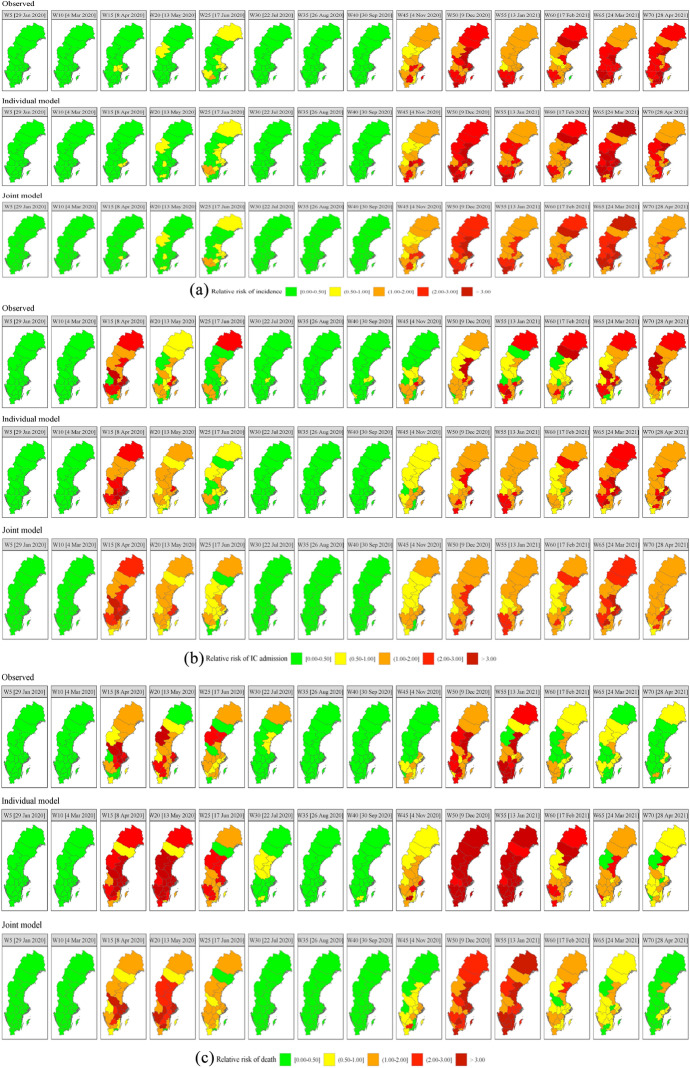
Observed and out-of-sample predicted relative risk using individual and joint modelling for **a** incidence, **b** IC admission, and **c** death for selected weeks (see note 24)

**Fig. 3:**
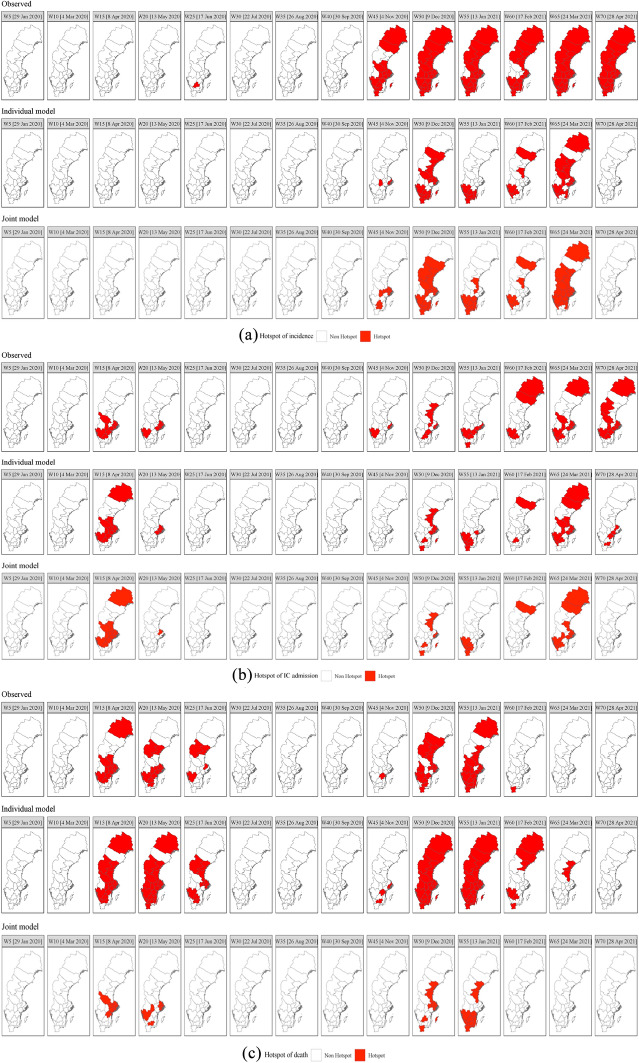
Observed and predicted relative risk hotspots for individual and joint modelling **a** incidence, **b** IC admission, and **c** death for selected weeks (given in note 24)

**Fig. 4:**
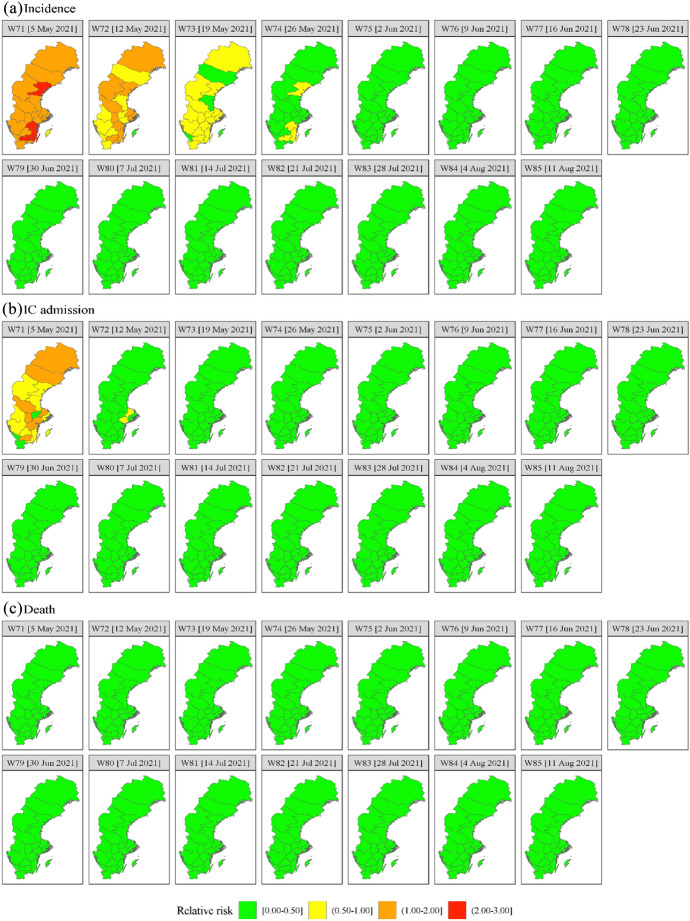
Predicted weekly relative COVID-19 risk of **a** incidence, **b** IC admission, and **c** death for W71-85 (5 May 2021—11 August 2021)

**Fig. 5:**
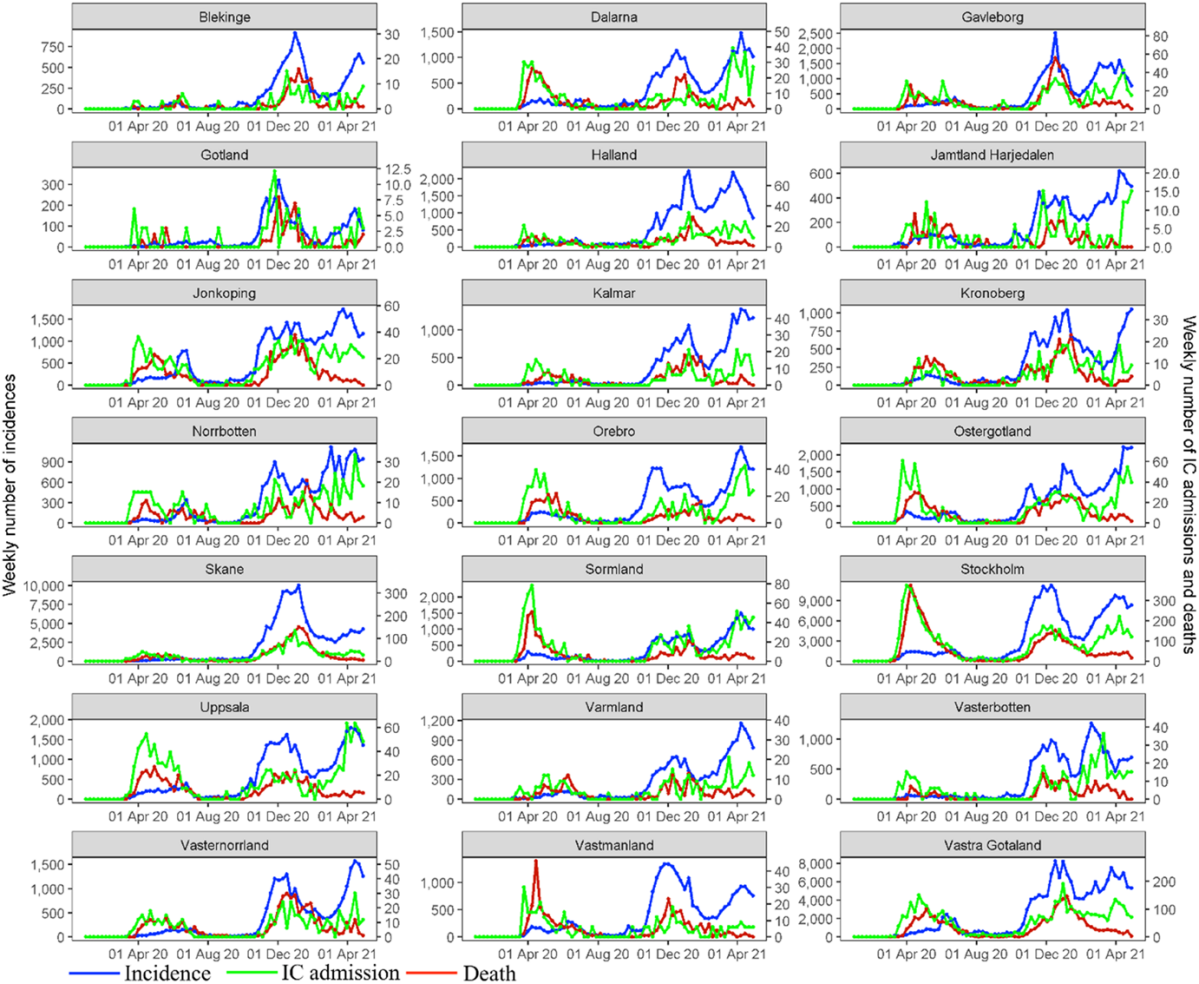
Weekly numbers of new incidences, IC admissions, and deaths per region. Note: The primary axis on the left-hand side denotes the weekly number of incidences, the secondary axis on the right-hand side the numbers of IC admissions and deaths

Figure 5 shows that in most regions there are three waves of incidences and IC admissions and two waves of deaths. The first wave of the three outcomes started in March 2020 and peaked in April 2020, especially in the Stockholm region (Owen 2021). The second wave started in September 2020 and peaked in December 2020. It had a rapid increase in the number of incidences, followed by an increase in IC admissions and deaths (Claeson and Hanson 2020). The third wave began in February 2020 and peaked in April 2021 .

**Table 2:** Posterior means of the fixed and random effects, their 95% credible intervals, the fractions of the variances, the impacts of the temporal shared effects, and the LCAR coefficients^a^

Fixed effects	Mean	Percentage change (%)	SE	95% credible interval
Intercept				
Incidence (${\alpha }^{y})$	− 2.471		0.180	(− 2.824; − 2.118)
IC admission (${\alpha }^{o})$	− 2.449		0.201	(− 2.843; − 2.055)
Death (${\alpha }^{z}$)	− 3.273		0.216	(− 3.697; − 2.849)
Slopes ^b^ of current and lagged $\mathrm{log}\left(\frac{{y}_{it}}{{E}_{it}^{y}}\right)$				
$\gamma_{11}$	0.148	14.8%	0.044	(0.061; 0.234)
$\gamma_{12}$	0.230	23.0%	0.044	(0.143; 0.317)
$\gamma_{21}$	0.139	13.9%	0.038	(0.063; 0.214)
$\gamma_{22}$	0.222	22.2%	0.040	(0.143; 0.301)
$\gamma_{23}$	0.133	13.3%	0.040	(0.056; 0.211)

Random effects	Mean	Fraction of the variance (FV) (%)^c^	SE	95% credible interval
Variance of the temporal specific effects				
Incidence (${\sigma }_{{\ddot{\phi }}^{y}}^{2})$	0.047	1.564%	0.010	(0.039; 0.054)
IC admission (${\sigma }_{{\ddot{\phi }}^{o}}^{2})$	0.022	0.726%	0.014	(0.017; 0.033)
Death (${\sigma }_{{\ddot{\phi }}^{z}}^{2})$	0.040	1.332%	0.012	(0.031; 0.050)
Variance of the temporal shared effect (${\sigma }_{\widetilde{\phi }}^{2})$	0.413	13.747%	0.071	(0.267; 0.633)
Impacts of the temporal shared weight			0.010	
Incidences (${\varrho }^{y}$)	1.000		0.000	(1.000; 1.000)
IC admission (${\varrho }^{o})$	1.064		0.105	(0.893; 1.297)
Death (${\varrho }^{z})$	1.117		0.162	(0.866; 1.482)
Variance of spatiotemporal interaction effect^d^				
Incidence (${\sigma }_{{\delta }^{y}}^{2})$	1.623	54.033%	0.076	(1.218; 1.924)
IC admission (${\sigma }_{{\delta }^{o}}^{2})$	0.344	11.465%	0.050	(0.236; 0.461)
Death (${\sigma }_{{\delta }^{z}}^{2})$	0.515	17.136%	0.050	(0.383; 0.666)
LCAR spatial autoregressive coefficient^e^				
Incidence (${\rho }^{y}$)	0.226		0.130	(0.154; 0.291)
IC admission (${\rho }^{o})$	0.426		0.325	(0.205; 0.665)
Death (${\rho }^{z})$	0.047		0.402	(0.019; 0.084)

^a^Dependent variables: log relative risk ($\mathrm{log}({\theta }_{it}^{h})$). The posterior means of the fixed and random effects in Table 2 are significant at 5%

^b^The coefficients are the elasticities of incidence with respect to IC admission and death. The coefficient of lagged $\mathrm{log}\left(\frac{{y}_{it}}{{E}_{it}^{y}}\right)$ for death is insignificant for lags larger than 2

^c^The contributions of the variances of the temporal specific random effect, space–time interaction effect and the temporal shared effect to the total variance of the three outcomes jointly. FV_*u*_ is defined as ${\mathrm{FV}}_{u}=\left({\sigma }_{u}^{2}/\sum_{u=1}^{U}{\sigma }_{u}^{2}\right)\times 100\%;u=1,\dots ,7$ with ${\sigma }_{u}^{2}=${${\sigma }_{{\ddot{\phi }}^{y}}^{2},{\sigma }_{{\ddot{\phi }}^{o}}^{2},{\sigma }_{{\ddot{\phi }}^{z}}^{2},{\sigma }_{\widetilde{\phi }}^{2},{\sigma }_{{\delta }^{y}}^{2},{\sigma }_{{\delta }^{o}}^{2}, {\sigma }_{{\delta }^{z}}^{2}$}

^d^The spatiotemporal interaction effect is the spatial effect $\otimes$ temporal effect with LCAR prior

^e^The prior corresponds to the spatial component of the spatiotemporal interaction effects

**Table 3:** Mean Absolute Error (MAE), Mean Absolute Percentage Error (MAPE), Root Mean Square Error (RMSE), and Pearson correlation coefficient (r) for joint and individual out-of-sample prediction for selected weeks^a^

Outcome	MAE		MAPE		RMSE		*r*	
	Individual	Joint	Individual	Joint	Individual	Joint	Individual	Joint
Incidence	0.204	0.173	0.351	0.312	0.346	0.315	0.963	0.963
IC admission	0.383	0.412	0.463	0.522	0.595	0.630	0.857	0.845
Death	1.145	0.388	1.724	0.663	1.846	0.645	0.917	0.917
Overall	0.578	0.324	0.846	0.499	1.137	0.551	0.827	0.906

^a^see note 24 for the selected weeks

**Table 4:** Misclassification rates of hotspot for incidence, (b) IC admission, and (c) death for selected weeks (given in note 24) for individual and joint modelling ^a^

Observed		Total observed	Individual		Joint		Misclassification rate (%)	
			Non-hotspot	Hotspot	Non-hotspot	Hotspot	Individual	Joint
Incidence	Non-Hotspot	181	181	0	181	0	24.150	20.068
	Hotspot	113	71	42	59	54		
	Total	294	252	42	240	54		
IC admission	Non-Hotspot	245	233	12	236	9	9.524	11.224
	Hotspot	49	16	33	24	25		
	Total	294	249	45	260	34		
Death	Non-Hotspot	236	199	37	235	1	12.585	10.544
	Hotspot	58	0	58	30	28		
	Total	294	199	95	265	29		
	Overall						15.420	13.946

^a^see note 24 for the selected weeks

**Table 5:** Deviance information criterion (DIC), Watanabe–Akaike information criterion (WAIC), marginal predictive likelihood (MPL), Pearson correlation ($$r)$$, mean absolute error (MAE) and root mean square error (RMSE)$${\widetilde{\mathrm{R}}}^{2})$$ $${\widetilde{\mathrm{R}}}^{2})$$ for 32 sub-models of the model in Eq. (8)^a^

Model	Likelihood	Temporal trend	DIC	WAIC	MPL	*r*	MAE	RMSE
V1	Poisson	RW1	104,992.020	145,409.230	− 66,877.300	0.968	56.140	15,063.460
		RW2	104,886.207	145,255.762	− 66,708.483	0.968	56.132	15,060.184
	NB	RW1	28,428.750	28,439.690	− 14,482.300	0.916	89.550	24,131.300
		RW2	28,397.561	28,403.162	− 14,399.921	0.902	97.601	26,057.890
V2	Poisson	RW1	90,815.940	132,076.870	− 60,021.120	0.968	54.610	15,041.190
		RW2	90,945.534	131,892.449	− 59,999.822	0.968	54.615	15,040.727
	NB	RW1	26,284.430	26,302.390	− 13,457.230	0.942	65.570	21,120.360
		RW2	26,227.391	26,237.468	− 13,418.912	0.944	64.001	20,222.566
V3	Poisson	RW1	103,206.660	144,375.740	− 66,077.860	0.968	56.100	15,041.610
		RW2	103,236.371	144,359.496	− 66,025.971	0.968	56.099	15,041.592
	NB	RW1	28,385.480	28,389.740	− 14,539.410	0.917	90.880	23,970.850
		RW2	28,340.891	28,345.446	− 14,466.966	0.909	96.604	25,250.818
V4	Poisson	RW1	23,708.730	24,525.150	− 20,557.460	1.000	1.930	199.890
		RW2	23,635.439	9.871E + 117	− 31,647.056	−0.004	8.685E + 17	3.830E + 21
	NB	RW1	24,648.720	24,611.000	− 20,219.480	1.000	4.410	771.100
		RW2	25,290.612	25,864.233	− 31,082.035	0.998	15.213	3,818.807
V5	Poisson	RW1	89,675.890	130,433.660	− 59,302.600	0.968	54.360	15,029.610
		RW2	89,744.467	130,273.250	− 59,296.622	0.968	54.366	15,029.688
	NB	RW1	NA	74,064.600	− 13,302.690	− 0.002	1.380E + 07	3.390E + 10
		RW2	26,051.149	26,066.773	-13,318.037	0.949	64.567	20,564.031
V6	Poisson	RW1	21,274.300	21,149.670	− 18,977.250	1.000	1.700	181.810
		RW2	21,371.412	1.107E + 21	− 30,242.464	1.000	2.591	316.789
	NB	RW1	21,555.360	21,423.550	− 18,960.270	1.000	1.910	214.020
		RW2	NA	5,686,254.429	− 30,151.749	0.067	1.640E + 149	7.209E + 152
V7	Poisson	RW1	23,724.450	24,556.130	− 20,484.570	1.000	1.970	205.380
		RW2	23,509.202	6.007E + 39	− 31,533.727	0.863	10.896	35,192.872
	NB	RW1	24,431.610	24,392.590	− 20,093.860	1.000	4.040	722.190
		RW2	25,378.709	26,070.806	− 31,137.748	0.998	15.321	3,799.430
V8	Poisson	RW1	21,275.900	21,148.050	− 18,942.850	1.000	1.690	180.100
		RW2	23,509.202	6.007E + 39	-31,533.727	0.863	10.896	35,192.872
	NB	RW1	24,431.610	24,392.590	− 20,093.860	1.000	4.040	722.190
		RW2	23,188.415	23,301.913	−30,011.364	0.998	12.945	3,363.680

^a^Note

V1: Model with spatial and temporal shared effects, current incidence and two of its time lags for IC admission and death. Time lags for death larger than two were insignificant

V2: V1 plus temporal specific effects

V3: V1 plus spatial specific effects

V4: V1 plus spatiotemporal interaction effects

V5: V1 plus temporal and spatial specific effects

V6: V1 plus temporal specific effects and spatiotemporal interaction effect

V7: V1 plus spatial specific effects and spatiotemporal interaction effect

V8: V1 plus spatial and temporal specific effects and spatiotemporal interaction effect

Several circumstances contributed to the high numbers of IC admissions and deaths during the first wave, notwithstanding the relatively low numbers of incidences. Despite the Swedish Public Health Agency's recommendations, testing was low or non-existent during the first wave (Folkhälsomyndigheten 2020; Froberg et al. 2021). In addition, mainly individuals admitted to hospitals were tested. Furthermore, there were frequent local outbreaks of asymptomatic healthcare workers in primary care institutions, home healthcare institutions, and palliative care institutions contributing to transmission, IC admission and death (Dillner et al. 2021; Froberg et al. 2021). Rapid local transmission was most pronounced in Stockholm where the bulk of COVID-19 deaths occurred during the initial outbreak (Folkhälsomyndigheten 2020).

The first step in the estimation procedure was model selection. Eight variants of model (8) were estimated and tested applying stepwise forward selection. The model variants differed by likelihood (Poisson and Negative Binomial), number of time lags of incidence ($y$) for IC admission ($o$) and death ($z$),^16^ structured spatial and structured temporal specific effects and structured spatiotemporal interaction.^17^ Structured spatial and structured temporal shared effects were included in all variants.

The base variant is V1 with the shared effects and the current and lagged incidences for IC admission and death. Variant V2 is V1 extended with the structured temporal specific effect while V3 and V4 are V1 extended with the structured spatial specific effect and the structured spatiotemporal interaction effect, respectively. (As we only consider structured effects (note 17). structured will be deleted below. In a similar vein, we just speak of spatiotemporal interaction or merely interaction as we only consider shared interaction). V5 is V1 plus the temporal and spatial specific effects. V6 and V7 are V2 and V3 extended with spatiotemporal interaction effect, respectively. V8 is V1 extended with temporal and spatial specific effects and spatiotemporal interaction effects. For each variant we considered sub-models by alternating the Poisson and the negative binomial likelihood and RW1 and RW2 trends. In total, we estimated 32 sub-models.^18^

We compared the fit of the 32 sub-models using the DIC and WAIC, and evaluated their predictive performance using the MPL, r, MAE, and RMSE, using leave-one-out^19^ cross-validation. The results are presented in Table 5, Appendix 2. As a rule of thumb, the best model is the one with the smallest DIC, WAIC, MAE, RMSE and the largest MPL and r. Table 5 shows that variant V6 with Poisson likelihood and RW1 trend performed best. It was selected for further analysis. The variant is denoted M6 below. Further analysis of M6 showed that the second time lag (${\gamma }_{23}$) of incidence had no significant effect on IC admission and was deleted (result available upon request). Furthermore, the precision of the spatial shared effect was infinite ($\infty$), corresponding to zero variance (result available upon request) indicating that it does not contribute to explaining the variation in the outcomes across space. We re-estimated the model without the spatial shared effect and the second time lag of incidence for IC admission. We assessed the performance of the revised M6 by examining the goodness of fit between the observed and predicted values. The correlation between the observed and predicted values of the three outcomes is above 0.90 (result available upon request) indicating that the model fits the data well (Bradley 2020). The estimation results are summarized in Table 2.

Below, we focus the discussion on the posterior means rather than on the credible intervals. Table 2 shows that the intercepts of incidence, IC admission, and death are ${\alpha }^{y}=-2.47;{\alpha }^{o}=-2.45, {\alpha }^{z}=-3.27$, respectively, giving the following mean relative risks: $\mathrm{exp}(-2.471)= 0.08$, $\mathrm{exp}\left(-2.45\right)=0.09$, and $\mathrm{exp}(-3.27)=0.04$, respectively. The means are close to zero, indicating that they do not explain much of the spatiotemporal risk variation. Consequently, the variation is mainly due to the other model components.

The regression slopes of lagged $\mathrm{log}\left(\frac{{y}_{it}}{{E}_{it}^{y}}\right)$ for all time lags have positive effects on the relative risk of IC admission and death. Specifically, the elasticities of lagged $\mathrm{log}\left(\frac{{y}_{it}}{{E}_{it}^{y}}\right)$ in the current and previous week for IC admission are 14.80% and 23.00%, respectively. For death the elasticities are 13.90%, 22.20%, and 13.30% for the current, previous and penultimate week, respectively. The elasticities for IC admission and death risk for the current and one-week lagged incidence are approximately the same with the strongest effects occurring for one-week lagged.

The posterior mean of the variance of a random effect represents its contribution to the variation of the risk estimate; the fraction its share in the total variation of the three estimates jointly. Table 2 shows that the posterior means of the variances of the random effects vary greatly, from 0.02 of the temporal effect for IC admission to 1.62 of the interaction effect for incidence risk. Table 2 furthermore shows that the variance of the temporal shared effect ($\widetilde{\phi }$) accounts for 13.75% of the joint random variation which is substantially smaller than the contribution of the joint spatiotemporal interaction effects ($54.03\%+11.47\%+17.14\%=82.63\%)$ but substantially larger than the contribution of the joint temporal effects (1.56% + 0.73% + 1.33% = 3.62%). The posterior means of the weights of the temporal shared effects of incidence, IC admission, and death are 1.00, 1.06, and 1.12, respectively, indicating that the temporal shared effect contributes similarly to the spatiotemporal variation of each disease outcome.

The correlations^20^ between the temporal shared effect and the temporal specific effect for incidence, IC admission, and death are 0.344, − 0.578, and 0.568, respectively. For incidence and death, the temporal shared effect and the specific temporal effect strengthen each other while for IC admission they work in opposite directions. The negative correlation for IC admission indicates that there were possibly different factors at work, notably intensive care capacity limitations necessitating that patients were transferred to hospitals in nearby regions to temporally free up space (Berger et al. 2022; Paterlini 2020; Winkelmann et al. 2022). The spatial autoregressive coefficients of the LCAR prior for IC admission, incidence, and death of 0.43, 0.23, and 0.05, respectively, are in line with this assumption. They indicate that for IC admission there was more spatial interaction than for the other outcomes.^21^

Figure 1 presents the estimated posterior means of the relative risk per outcome for 21 regions for selected weeks based on M6 with parameters listed in Table 2.^22^ The weeks are chosen to highlight the shifts in the posterior means of three outcomes. A relative risk greater than one means that the corresponding posterior mean is larger than the overall average across space and time.

Figure 1(a) shows that the estimated relative risk of COVID-19 incidence in all regions was low during the first 13 weeks W1-W13 (1 January 2020–25 March 2020) (estimated relative risk < 0.50, in green). From W14 (1 April 2020) onward, it began to increase, starting at medium intensity (estimated relative risk 0.5–1.0, yellow) in two southern regions, and subsequently spreading to several western and northern regions. The increase in incidence coincided with an increase in testing, although with local variation (Florida and Mellander 2021; Folkhalsomyndigheten 2020; Pashakhanlou 2021). For instance, in Stockholm, substantial upscaling of testing capacity happened during the second half of the first wave (Fredriksson and Hallberg 2021; Roden 2020). During the weeks W24-W26 (10 June 2020 24 June 2020), there were two high-risk regions (estimated relative risk 1–2, orange) in the southwest and medium risk regions (estimated relative 0.5–1.0, yellow) in the north and the east. During the next week the high-risk regions disappeared and the number of medium risk regions declined. Between W27-W43 (1 July 2020–21 October 2020) there were no high-risk regions and the number of medium risk regions declined further. During W43 (21 October 2020) the next outbreak started in two regions in the south with relatively high intensity (orange). Next, the outbreak began spreading to the majority of the regions. Between W45–W70 (4 November 2020–28 April 2021), more than 80% of Sweden's regions were high risk regions. Some regions in the north had extremely high risk (relative risk > 3).

Figure 1(b) shows that the estimated relative risk of IC admission was low (green) in all regions during the first 10 weeks. It began to rise in W11 (11 March 2020), with moderate intensity (yellow) in the south-east. Next, it started quickly to spread to more than 80% of the regions until W17 (22 April 2020). Between W18-W43 (29 April 2020–21 October 2020), the estimated IC admission risk gradually started decreasing. From W44 (28 October 2020) onwards, the estimated risk of IC admission began to rise again until W70 (28 April 2021). Over 80% of the regions were high risk regions during that period, several of them extremely high-risk regions, particularly in the north.

Figure 1(c) presents the estimated spatiotemporal distribution of the relative risk of COVID-19 death. During the first 11 weeks W1–W11 (1 January 2020–11 March 2020) it was low in all regions (green) but began to turn moderate (yellow) in the south-east and Stockholm followed by a quick proliferation (orange) to more than 80% of the regions until W21 (20 May 2020. From W22-W43 (27 May 2020–21 October 2020), the estimated death risk gradually decreased everywhere but started to rise again in W43 (21 October 2020) to reach a peak in W53 (30 December 2020) with over 80% of the regions high risk regions, and several of them extremely high risk regions, particularly in the southeast and north. With the exception of the north, the estimated death risk continuously decreased from W68 (14 April 2021) until the end of W70 (April 2021).

The estimated relative risks for the three outcomes show similarities but also differences (see also Fig. 3 in Online Resource (1). The relative IC admission risk and relative death risk are strongly correlated across space and time, with correlation greater than 0.70. The correlations between the relative incidence risk on the one hand and the relative IC admission risk and the relative death risk on the other are greater than 0.50 but smaller than the correlation between the relative IC admission risk and death risk, notably during the early stages of the pandemic. We hypothesize that this is related to the slow start of testing and recording of the three outcomes. As mentioned above, testing was low or non-existent during the first wave depressing the number of incidences (Folkhalsomyndigheten 2021; Froberg et al. 2021). Particularly, persons with only mild symptoms were recommended not to seek medical care or visit hospitals and thus forewent testing leading to underestimation of incidence. Another factor contributing to the relatively low correlation between the relative incidence risk and the relative IC admission risk is that during the initial stages, a large proportion of the infection occurred at nursing homes where many infected elderly died before they were admitted to IC (Nordström et al. 2021).^23^

## Prediction of the joint COVID-19 outcomes using M6, 5 May 2021–11 August 2021

The estimated M6 is used to generate weekly forecasts for the three relative risks for 15 weeks ahead from W71-W85 (5 May 2021–11 August 2021). Before doing so, we analysed the predictive performance of M6 by out-of-sample prediction for selected weeks.^24^ At the same time, we compared joint modelling with individual modelling. Individual models for three relative risks have the same model specification as M6 but without the temporal shared component. In addition, they are estimated individually. The observed and joint and individual model relative risks predictions are presented in Fig. 2.

Figure 2 shows that the joint model quite accurately predicts the three kinds of relative risk and outperforms the individual approach, except for IC admission. This also follows from the Mean Absolute Error (MAE), Mean Absolute Percentage Error (MAPE), Mean Square Error (MSE), and the Pearson correlation coefficient (r) in Table 3.^25^ The table shows that compared to the individual approach the joint approach has smaller MAE, MAPE, and RMSE and larger $r$ for incidence and death relative risk $.$ For IC admission relative risk the MAE, MAPE and RMSE are larger for the joint approach than for the individual models.

Individual and joint prediction are further evaluated by considering the identification of spatiotemporal hotspots, (discussed in Sect. 2) with threshold $c=1$ and the cut-off value $\gamma =0.90.$ The spatiotemporal pattern of observed ($\mathrm{Pr}\left({\mathrm{SER}}_{it}^{h}>1|\mathbf{g}\right)>.90)$ versus predicted ($\mathrm{Pr}\left({\widehat{\theta }}_{it}^{h}>1|\mathbf{g}\right)>.90$) hotspots is presented in Fig. 3 while the misclassification rates are presented in Table 4.

Table 4 shows that the misclassification rates of both approaches are lower than 30% indicating a good performance (Limam et al. 2004). The joint approach clearly outperforms the individual approach, except for IC admission which is probably related to the intensive care capacity problems and recording issues discussed in see Sect. 3.

The misclassifications for joint modelling concern especially incidence and IC admission risk and to a less extent death risk. In the case of incidence risk, 59 observed hotspots are incorrectly predicted to be non-hotspots while 235 spatiotemporal units were correctly classified (i.e. 181 observed hotspots and 54 observed non-hotspots were correctly predicted). For IC admission 9 observed non-hotspots were incorrectly classified as hotspots and 24 observed hotspots were predicted as non-hotspots. For death 1 observed non-hotspot was incorrectly classified as hotspot and 30 observed hotspots were incorrectly classified as non-hotspot. Hence, the problem is the misclassification of hotspots as non-hotspots. Note that misclassification increases for increasing cut-off values $\gamma$ for fixed threshold $c$ or, alternatively, for increasing $c$ for fixed $\gamma$ (Jaya and Folmer 2021a). As a consequence, if for policy reasons the emphasis is on the correct identification of hotspots, $c$ or $\gamma$ could be lowered to reduce the misclassification rate of hotspots.

Finally, we present the spatiotemporal predictions of the relative risks of the three outcomes in Fig. 4 for W71–W85 (5 May 2021–11 August 2021) using M6.

Figure 4 shows that in W71 (5 May 2021) the predicted relative risk for incidence is high (> 1) for the entire country and extremely high (> 2) in some regions in the south-east and middle-east. The following week the regions with extremely high relative incidence risk have disappeared while a substantial number of high risk regions have become moderately risky. In W73 (19 May 2021) the entire country has become moderately risky while for some regions the relative risk has dropped below 0.5. In W74 (26 May 2021) the relative incidence risk has dropped below 0.5 almost everywhere except for some regions in the southeast and the middle east with a relative risk between 0.5 and 1. For the remainder of the prediction period, the relative incidence risk is less than 0.5 everywhere.

For IC admission the relative risk is high in the north, in mid-Sweden and in a region in the southeast in W71 (5 May 2021). It is moderately risky for the remainder of the country while for some regions it is below 0.5. For the remainder of the prediction period W73-W85 (19 May 2021–11 Augustus 2021). IC admission is predicted to be below 0.5 everywhere. The relative death risk for the entire country for the entire prediction period is predicted to be below 0.5. Finally, no hotpots are predicted for the three outcomes for the entire country for the entire prediction period for $c$ = 1 and $\gamma = 0.90$.^26^

## Conclusions

This paper presents a spatiotemporal Bayesian hierarchical model for the joint prediction of the relative risk of the COVID-19 outcomes incidence, intensive care admission and death. The model takes into account common and specific temporal patterns of the outcomes, thus capturing similarities and differences in the spatiotemporal prediction distribution of the relative risk associated with each outcome. Following Chu (2021), Newalla et al. (2020) and Scobie et al. (2021), we argued that joint prediction reduces biased estimation of the parameters of the constituting individual equations, thus improving forecasting and mapping. The application is in line with this proposition. It showed that for incidence risk and death risk the prediction of hotspots by the joint approach outperforms the predictions by the individual approaches.

The joint approach presented here applies a pure model that captures the spatiotemporal developments of the dependent variables in terms of structured and unstructured spatial and temporal random effects and their interaction. It thus accounts for the impacts of the covariates on the dependent variables indirectly, namely in terms of spatiotemporal trends. The pure model is “data scarce” because it only requires observations on the population at risk and the dependent variable per spatiotemporal unit. In the application, the spatial unit for both variables is the region, the timeframe for the population at risk is 15 months and for the 3 outcomes a week. The data configuration allows frequent updating and makes it suitable for weekly prediction. For COVID-19, but also for other infectious diseases, this is a basic requirement for the health authorities, hospitals and funeral enterprises to take suitable action, such regional or national coordination of hospitalization and IC admission, particularly for the short term.

In the application, we predicted the relative risk of COVID-19 incidence, IC admission, and death in Sweden from January 1, 2020 to May 4, 2021. The analysis revealed a common temporal pattern between the three COVID-19 risks. The finding is consistent with Scobie et al. (2021). The joint model was applied to forecast the incidence risk, IC admission risk and death risk for the period after the sample period, i.e. from 5 May 2021 to 11 August 2021. The model forecasted a significant decline in the relative risk of all three outcomes and no hotspots.

To conclude this section, we briefly discuss a causal model containing covariates as an alternative model to the pure model to generate COVID-19 predictions, viz. Olmo and Sanso-Navarro (2021). This paper uses a conventional econometric approach to predict the number of COVID-19 incidences while applying a Bayesian averaging framework to accommodate the large set of potential covariates. The model is instrumental to evaluating the critical covariates of COVID-19 incidence and explaining differences in the number of new confirmed cases across neighbourhoods for a given time period. However, because the model is based on static (time-invariant) covariates, forecasting spatiotemporal variation in COVID-19 outcomes is not possible, as shown by Lim et al. (2021) and Wen et al. (2018). A shortcoming of our model on the other hand is that it does not explicitly identify the socioeconomic and environmental factors driving the spatiotemporal variation of the disease outcomes. However, if the covariates are measured for the same space–time units as the outcomes, extension of the pure model to a causal model can be achieved straightforwardly (Jaya and Folmer 2021b, 2022a under review, 2022b).

Our approach furthermore complements the Olmo and Sanso-Navarro (2021) approach in that it explicitly considers spatiotemporal interaction. The inclusion of this variable in a prediction model for an infectious disease such as COVID-19 is basic as the intensity of infectious diseases tends to move in space and time. The case study presented in the Sects. 3 and 4 showed that for incidence and death the variance of the interaction effect is by far the most important component of the total variance and for IC capacity the next largest (see Table 2). The spatiotemporal interaction effect of COVID-19 transmission among regions is critical to formulating pandemic policies (Liu et al. 2021; Wang et al. 2021; Wu et al. 2021).

Finally, we note that the paper is limited in scope in that it presents spatiotemporal predictions of the overall relative risks of incidence, IC admission and death. Although this is an interesting and policy relevant objective, disaggregation by sub-populations according to inter alia age, sex, profession, health status, travel behaviour are highly policy relevant as the three kinds of relative risk have been found to vary across sub-populations (Newalla et al. 2020). In addition, spatial disaggregation is desirable as the Swedish regions are very heterogeneous.

### Electronic supplementary material

Below is the link to the electronic supplementary material.

Supplementary file1 (DOCX 1217 kb)

## Data Availability

Available upon request.

## Appendix 1

Spatiotemporal distribution of the disease outcomes

See Fig. 5

## Appendix 2

Model comparison

See Table 5

## Footnotes

1 One reason for measurement error of the number of incidences in Sweden was that, initially, the testing capacity was insufficient and therefore concentrated in regions with high numbers of IC admissions and deaths from COVID-19.

2 In contrast to most other European countries, Sweden did not implement lockdowns and school closures (Florida and Mellander 2021; SOU 2021a,b). Whether or not this affected spatiotemporal variation is a matter of further investigation. The impact of the omission on prediction by means of the pure model, however, is not significantly different from the impacts of other unobserved variables, as it is captured by spatial and temporal random effects and their interaction. See below and Sect. 2.

3 The pure model is also known as the random intercept or null model (Rouamba et al. 2020; Waller and Carlin 2010).

4 Note that pure models can be used to generate hypotheses about the causes of the disease, notably identification possible risk factors (Huque et al. 2016; Jaya and Folmer 2021a; Wakefield 2007).

5 The latent correlation between the random effects and the covariates is known as spatiotemporal confounding (Azevedo et al. 2020; Adin et al. 2022; Clayton et al. 1993; Jaya and Folmer 2022a, under review; Johnston et al. 2018).

6 Jaya and Folmer (2022a, under review) shows that a pure model can be straightforwardly expanded to a causal model by including covariates, in addition to the spatiotemporal random effects and their interaction.

7 To account for the population structure such as the age or sex distribution, direct or indirect standardization can be applied (Sahu and Böhning 2021; Waller and Gotway 2004).

8 The objective of the paper is to generate spatiotemporal forecasts of the COVID-19 death risk as such (in addition to incidences and IC admissions risk), independent of IC admission or other treatments. The objective is not the prediction of the spatiotemporal death risk among hospitalized patients. Of course, this is also a valid objective and policy relevant, but different from forecasting spatiotemporal death risk in general. For the latter, only the incidence risk is needed. Spatiotemporal differences in IC admission risk are not needed for this as their their impacts are captured by the spatiotemporal random effects and their interaction.

9 The unstructured spatial effect and the unstructured temporal effect are not decomposed into specific and shared effects because they do not account for the spatiotemporal patterns but for spatiotemporal heterogeneity (i.e., deviations from the spatiotemporal trends) (Gomez-Rubio et al. 2019; Knorr-Held and Best 2001).

10 Given the linear predictors ${\eta }_{it}^{h}$for$h=y,o,z,$ the probabilities of the numbers of incidences, IC admissions, and deaths are conditionally independent of one another as ${\eta }_{it}^{h}$ accounts for the dependency between the outcomes (Niekerk et al. 2021). Therefore, the joint probability distribution of the numbers of incidences, IC admissions and deaths given ${\eta }_{it}^{h}$is defined as$p\left({y}_{it},{o}_{it},{z}_{it}|{\eta }_{it}^{h}\right)=p\left({y}_{it}|{\eta }_{it}^{h}\right)p\left({o}_{it}|{\eta }_{it}^{h}\right)p\left({z}_{it}|{\eta }_{it}^{h}\right)$.

11 The Leroux CAR is the equivalent of the spatial error model (SEM) in spatial econometrics (Bivand et al. 2013).

12 Both priors belong to the class of Markov random fields.

13 Unstructured spatial (temporal) random effects are assumed to be independent (Osei and Stein 2019).

14 The precision matrix **Q**(${\varvec{\Psi}}$) is the inverse of the covariance matrix ${\varvec{\Sigma}}\left({\varvec{\Psi}}\right)$. The use of the precision matrix rather than the covariance matrix is useful (or even required) in many spatiotemporal applications as it allows for a sparse representation, typically leading to less computing time and memy complexity (Sidén et al. 2018).

15 Compared to the Markov Chain Monte Carlo method which uses a simulation approach to the full conditional posterior distribution, the INLA approach uses numerical approximation to the posteriors of interest using Laplace approximation (see Tierney and Kadane 1986).

16 According to Strålin et al. (2020), IC admission is concentrated within two weeks after COVID-19 diagnosis. Hence, for IC admission we considered current incidence and incidence lagged for two weeks. For death we considered time lags until the coefficients were insignificant.

17 We also considered the inclusion of unstructured spatial and temporal effects. We found that the models with unstructured effects had worse evaluation criteria than the models in Table 2 (Appendix 2) and were not considered further. The results are available upon request.

18 The estimates of the models with the other types of interaction are available upon request.

19 Leave-one-out cross-validation is a learning algorithm with only one observation serving as the testing set and all other observations as the training set (Sammut and Webb 2010).

20 The temporal patterns of the temporal specific and shared effects can be found in Fig. A1 in Online Resource 1.

21 The interaction effects maps can be found in Fig. A2 in Online resource 1.

22 The complete set of spatiotemporal disease risk per outcome risk per region is available in Fig. A3 in Online Resource 1.

23 This is highlighted and further discussed in SOU (2021a, b). Particularly, although the municipalities are responsible for the nursing homes, they do not have the right to hire doctors which negatively affected admission to intensive care. However, in the death certificate, COVID-19 was registered as the cause of death. In addition, there were also cases of correctly diagnosed incidences but that were not admitted to intensive care due to, for instance, bad physical shape of the patient.

24 The selected testing weeks were: W5 (29 Jan 2020), W10 (4 Mar 2020), W15 (8 Apr 2020), W20 (13 May 2020), W25 (17 Jun 2020), W30 (22 Jul 2020), W35 (26 Aug 2020), W40 (30 Sep 2020), W45 (4 Nov 2020), W50 (9 Dec 2020), W55 (13 Jan 2021), W60 (17 Feb 2021), W65 (24 Mar 2021), and W70 (28 Apr 2021). Out -of– sample means here that the testing sample and training sample fall within the observation period.

25 The MAE, MAPE, MSE and r for relative risk $h$, are defined as (Dong et al. 2019): $$\begin{aligned} {\text{MAE}}^{h} & = \frac{1}{{n\tilde{T}}}\sum\nolimits_{{\tilde{t} = 1}}^{{\tilde{T}}} {\sum\limits_{{i = 1}}^{n} {\left| {{\text{SER}}_{{i\tilde{t}}}^{h} - \theta _{{i\tilde{t}}}^{h} } \right|} } {\text{, }} \\ {\text{MAPE}}^{h} & = \frac{1}{{n\tilde{T}}}\sum\limits_{{\tilde{t} = 1}}^{{\tilde{T}}} {\sum\limits_{{i = 1}}^{n} {\left| {\frac{{{\text{SER}}_{{i\tilde{t}}}^{h} - \theta _{{ii\tilde{t}}}^{h} }}{{{\text{SER}}_{{i\tilde{t}}}^{h} }}} \right|} } {\text{, }} \\ {\text{RMSE}}^{h} & = \sqrt {\frac{1}{{n\tilde{T}}}\sum\limits_{{\tilde{t} = 1}}^{{\tilde{T}}} {\sum\limits_{{i = 1}}^{n} {\left( {{\text{SER}}_{{i\tilde{t}}}^{h} - \theta _{{i\tilde{t}}}^{h} } \right)^{2} } } } ,\;{\text{and}} \\ {\text{ }}r^{h} & = \frac{{\sum\nolimits_{{\tilde{t} = 1}}^{{\tilde{T}}} {\sum\nolimits_{{i = 1}}^{n} {\left( {{\text{SER}}_{{i\tilde{t}}}^{h} - \overline{{{\text{SER}}}} _{{i\tilde{t}}}^{h} } \right)} } \left( {\theta _{{i\tilde{t}}}^{h} - \bar{\theta }_{{i\tilde{t}}}^{h} } \right)}}{{\sqrt {\sum\nolimits_{{\tilde{t} = 1}}^{{\tilde{T}}} {\sum\nolimits_{{i = 1}}^{n} {\left( {{\text{SER}}_{{i\tilde{t}}}^{h} - \overline{{{\text{SER}}}} _{{i\tilde{t}}}^{h} } \right)^{2} } } \sum\nolimits_{{\tilde{t} = 1}}^{{\tilde{T}}} {\sum\nolimits_{{i = 1}}^{n} {\left( {{\text{SER}}_{{i\tilde{t}}}^{h} - \bar{\theta }_{{i\tilde{t}}}^{h} } \right)^{2} } } } }} \\ \end{aligned}.$$ with $\tilde{t} = 1,2..14$ corresponding to e selected weeks in note 24. In the case of the MAPE, we removed zero $SER_{{i\tilde{t}}}^{h} s$ to avoid unidentified values. Ceteris paribus, the lower the MAE, MAPE and RMSE and the higher *r*, the better the predictive performance.

26 Predictions of exceedance probability of the three outcomes are available upon request.

## Code availability

Available upon request.

## References

[CR1] Abente LG, Aragonés N, García-Pérez J, Fernández NP (2018). Disease mapping and spatio-temporal analysis: importance of expected-case computation criteria. Geospat Health.

[CR2] Adin A, Goicoa T, Hodges J, Schnell P, Ugarte M (2022). Alleviating confounding in spatio-temporal areal models with an application on crimes against women in India. Stat Model.

[CR3] Agarwal D, Gelfand A, Citron-Pousty S (2002). Zero-inflated models with application to spatial count data. Environ Ecol Stat.

[CR4] Aleman V, Fernan E, Varon D, Surani S, Gathe J, Varon J (2020). Socioeconomic disparities as a determinant risk factor in the incidence of COVID-19. Chest.

[CR5] Arani HZ, Manshadi GD, Atashi HA, Nejad AR, Ghorani SM, Abolghasemi S, Bahrani M, Khaledian H, Savodji PB, Hoseinian M, Bejandi AK, Abolghasemi S (2021). Understanding the clinical and demographic characteristics of second coronavirus spike in 192 patients in Tehran Iran: a retrospective study. PLoS One.

[CR6] Azevedo D, Bandyopadhyay D, Prates M, Abdel-Salam AS, Garcia D (2020). Assessing spatial confounding in cancer disease mapping using R. Cancer Rep.

[CR7] Azuma K, Yanagi U, Kagi N, Kim H, Ogata M, Hayashi M (2020). Environmental factors involved in SARS-CoV-2 transmission: effect and role of indoor environmental quality in the strategy for COVID-19 infection control. Environ Health Prev Med.

[CR8] Balamchi S (2021) Spatial modeling of repeated events. Winnipeg Manitoba Department of Statistics University of Manitoba

[CR9] Berger E, Winkelmann J, Eckhardt H, Nimptsch U, Panteli D, Reichebner C, Rombey T, Busse R (2022). A country-level analysis comparing hospital capacity and utilisation during the first COVID-19 wave across Europe. Health Policy.

[CR10] Berk R, MacDonald J (2008). Overdispersion and poisson regression. J Quant Criminol.

[CR11] Besag J, York J, Mollié A (1991). Bayesian image restoration with two applications in spatial statistics. Ann Inst Stat Math.

[CR12] Bivand R, Pebesma E, Gómez-Rubio V (2013). Applied spatial data analysis with R.

[CR13] Bivand R, Gomez-Rubio V, Rue H (2015). Spatial data analysis with R-INLA with some extensions. J Stat Softw.

[CR14] Blangiardo M, Cameletti M (2015). Spatial and spatio-temporal Bayesian models with R – INLA.

[CR15] Borchering R, Viboud C, Howerton E, Smith C (2021). Modeling of future COVID-19 cases hospitalizations and deaths by vaccination rates and nonpharmaceutical intervention scenarios—United States April–September 2021. Morb Mortal Wkly Rep.

[CR16] Bradley J (2020) Joint Bayesian analysis of multiple response-types using the hierarchical generalized transformation model. Bayesian Anal 1–38

[CR17] Brett T, O’Dea E, Marty E, Miller P, Park A, Drake J, Rohani P (2018). Anticipating epidemic transitions with imperfect data. PLoS Comput Biol.

[CR18] Briz-Redón Á, Serrano-Aroca Á (2020). A spatio-temporal analysis for exploring the effect of temperature on COVID-19 early evolution in Spain. Sci Total Environ.

[CR19] Cerqua A, Letta M (2022). Local inequalities of the COVID-19 crisis. Reg Sci Urban Econ.

[CR20] Chan H, Skali A, Savage D, Stadelmann D, Torgler B (2020). Risk attitudes and human mobility during the COVID 19 pandemic. Sci Rep.

[CR21] Choo L, Walker S (2008). A new approach to investigating spatial variations of disease. J R Stat Soc.

[CR22] Chu JA (2021). Statistical analysis of the novel coronavirus (COVID-19) in Italy and Spain. PLoS One.

[CR23] Claeson M, Hanson S (2020). COVID-19 and the Swedish enigma. Lancet.

[CR24] Clayton DG, Bernardinelli L, Montomoli C (1993). Spatial correlation in ecological analysis. Int J Epidemiol.

[CR25] Congdon P (2021). Mid-epidemic forecasts of COVID-19 cases and deaths: a bivariate model applied to the UK. Interdiscip Perspect Infect Dis.

[CR26] Dey D, Chen MH, Chang H (1997). Bayesian approach for nonlinear random effects models. Biometrics.

[CR27] Dillner J, Elfström K, Blomqvist J, Engstrand L, Uhlén M, Eklund C (2021). High amounts of SARS-CoV-2 precede sickness among asymptomatic health care workers. J Infect Dis.

[CR28] Downing A, Forman D, Gilthorpe M, Edwards K, Manda S (2008). Joint disease mapping using six cancers in the Yorkshire region of England. Int J Health Geogr.

[CR29] Elezkurtaj S, Greuel S, Ihlow J, Michaelis E, Bischoff P, Kunze C (2021). Causes of death and comorbidities in hospitalized patients with COVID-19. Sci Rep.

[CR30] Eslami H, Jalili M (2020). The role of environmental factors to transmission of SARS-CoV-2 (COVID-19). AMB Expr.

[CR31] Florida R, Mellander C (2021) The geography of COVID 19 in Sweden. Ann Reg Sci 1–26 10.1007/s00168-021-01071-0 PMC8299438 34316091

[CR32] Folkhälsomyndigheten (2020) The infection fatality rate of COVID-19 in Stockholm—technical report Sweden: Public health agency of Sweden. Available at: https://www.folkhalsomyndigheten.se/contentassets/53c0dc391be54f5d959ead9131edb771/infection-fatality-rate-covid-19-stockholm-technical-report.pdf. Accessed 26 Nov 2021

[CR33] Folkhälsomyndigheten (2021) December 2 COVID-19 testing. Available at: https://www.folkhalsomyndigheten.se/the-public-health-agency-of-sweden/communicable-disease-control/covid-19/covid-19-testing/. Accessed 10 Dcember 2021

[CR201] Folkhalsomyndigheten (2020) The infection fatality rate of COVID-19 in Stockholm – Technical report Sweden: Public Health Agency of Sweden. Available at: https://www.folkhalsomyndighetense/contentassets/53c0dc391be54f5d959ead9131edb771/infection-fatality-rate-covid-19-stockholm-technical-report.pdf. Accessed 26 Nov 2021

[CR34] Fredriksson M, Hallberg A (2021). COVID-19 testing in Sweden during 2020–split responsibilities and multi-level challenges. Front Public Health.

[CR35] Froberg M, Hassan S, Pimenoff V, Akterin S, Lundgren K, Elfstrom K, Dillner J (2021). Risk for SARS-CoV-2 infection in healthcare workers outside hospitals: a real-life immuno-virological study during the first wave of the COVID-19 epidemic. PLoS ONE.

[CR36] Gelman A (2006). Prior distributions for variance parameters in hierarchical models. Bayesian Anal.

[CR120] Gómez-Rubio V (2020). Bayesian inference with INLA.

[CR37] Gomez-Rubio V, Palmı-Perales F, Lopez-Abente G, Ramis-Prieto R, Fernandez-Navarro P (2019). Bayesian joint spatio-temporal analysis of multiple diseases. SORT.

[CR38] Hawkins R, Charles E, Me J (2020). Socio-economic status and COVID-19-related cases and fatalities. Public Health.

[CR39] Huque M, Anderson C, Walton R, Ryan L (2016). Individual level covariate adjusted conditional autoregressive (indiCAR) model for disease mapping. Int J Health Geogr.

[CR40] Iddrisu A-K, Alhassan A, Ami N (2018). Investigating spatio-temporal pattern of relative risk of tuberculosis in Kenya using Bayesian hierarchical approaches. J Tuberc Res.

[CR41] IHME (2021). Modeling COVID-19 scenarios for the United States. Nat Med.

[CR42] Jaya IGNM, Folmer H (2020). Bayesian spatiotemporal mapping of relative Dengue disease risk in Bandung Indonesia. J Geogr Syst.

[CR43] Jaya IGNM, Folmer H (2021). Bayesian spatiotemporal forecasting and mapping of COVID-19 risk with application to West Java Province Indonesia. J Reg Sci.

[CR44] Jaya IGNM, Folmer H (2021). Identifying spatiotemporal clusters by means of agglomerative hierarchical clustering and Bayesian regression analysis with spatiotemporally varying coefficients: methodology and application to dengue disease in Bandung, Indonesia. Geogr Anal.

[CR45] Jaya IGNM, Folmer H (2022). Spatiotemporal high-resolution prediction and mapping: methodology and application to dengue disease. J Geogr Syst.

[CR46] Jaya IGNM, Folmer H, Ruchjana BN, Kristiani F, Yudhie A, Jackson R, Schaeffer P (2017). Modeling of infectious diseases: a core research topic for the next hundred years. Regional research frontiers - methodological advances regional systems modeling and open sciences.

[CR47] Jaya IGNM, Folmer H (2022a) Does the inclusion of trending and spatially confounded covariates improve the forecasting accuracy of spatiotemporal models? A simulation study of univariate and causal forecasting models. (Under review)

[CR48] Johnston R, Jones K, Manley D (2018). Confounding and collinearity in regression analysis: a cautionary tale and an alternative procedure, illustrated by studies of British voting behaviour. Qual Quant.

[CR49] Karmakar M, Lantz P, Tipirneni R (2021). Association of social and demographic factors with COVID-19 incidence and death rates in the US. JAMA Netw Open.

[CR50] Kazembe L (2007). Spatial modelling and risk factors of malaria incidence in northern Malawi. Acta Trop.

[CR51] Knorr-Held L (2000). Bayesian modeling of inseparable space-time variation in disease risk. Stat Med.

[CR52] Knorr-Held L, Best N (2001). A shared component model for detecting joint and selective clustering of two diseases. J R Stat Soc.

[CR53] Last J (2001). A dictionary of epidemiology.

[CR54] Lawson A (2010). Hotspot detection and clustering: ways and means. Environ Ecol Stat.

[CR55] Lawson A, Lee D, Rao A, Pyne S, Rao C (2017). Bayesian disease mapping for public health. Handbook of statistics disease model and public health part A.

[CR56] Lawson A, Rotejanaprasert C (2014). Childhood brain cancer in Florida: a Bayesian clustering approach. Stat Public Policy.

[CR57] Lee D (2011). A comparison of conditional autoregressive models used in Bayesian disease mapping. Spat Spatiotemporal Epidemiol.

[CR58] Leroux B, Lei X, Breslow N, Halloran M, Berry D (2000). Estimation of disease rates in small areas: a new mixed model for spatial dependence. Statistical models in epidemiology the environment and clinical trials.

[CR59] Lesaffre E, Lawson A (2012). Bayesian biostatistics.

[CR60] Lewsey J, Thomson W (2004). The utility of the zero-inflated Poisson and zero-inflated negative binomial models: a case study of cross-sectional and longitudinal DMF data examining the effect of socio-economic status. Commun Dent Oral Epidemiol.

[CR61] Lim B, Arık SÖ, Loeff N, Pfister T (2021). Temporal fusion transformers for interpretable multi-horizon time series forecasting. Int J Forecast.

[CR62] Limam M, Diday E, Wi S, Aachen H, Karlsruhe W, Rome M (2004). Probabilistic allocation of aggregated statistical units in classification trees for symbolic class description. Studies in classification data analysis and knowledge organisation.

[CR63] Liu J, Liao X, Qian S, Yuan J, Wang F, Liu Y, Wang Z, Wang F-S, Liu L, Zhang Z (2020). Community transmission of severe acute respiratory syndrome Coronavirus 2 Shenzhen China 2020. Emerg Infect Dis.

[CR64] Liu L, Hu T, Bao S, Wu H, Peng Z, Wang R (2021). The spatiotemporal interaction effect of covid-19 transmission in the United States. Int J Geoinf.

[CR65] Lopez-Quılez A, and Munoz F, 2009 Review of spatio-temporal models for disease mapping The EUROHEIS2 project

[CR66] Ma Y, Zhao Y, Liu J, He X, Fu S, Yan J, Niu J, Zhou J, Lou B (2020). Effects of temperature variation and humidity on the death of COVID-19 in Wuhan China. Sci Total Environ.

[CR67] Mahaki B, Mehrabi Y, Kavousi A, Schmid V (2018). Joint spatio-temporal shared component model with an application in Iran cancer data. Asian Pac J Cancer Prev.

[CR68] Martinez-Beneito M, Botella-Rocamora P (2019). Disease mapping from foundations to multidimensional modeling.

[CR69] Martins R, Silva G, Andreozzi V (2016). Bayesian joint modeling of longitudinal and spatial survival AIDS data. Stat Med.

[CR70] Mutair A, Mutairi A, Alhumaid S, Abdullah S, Zaidi A, Rabaan A, Al-Omari A (2021). Examining and investigating the impact of demographic characteristics and chronic diseases on mortality of COVID-19: retrospective study. PLoS ONE.

[CR71] Nature (2020) COVID-19 and human behaviour nature. Available at: https://www.nature.com/collections/gdjdibibfg. Accessed 10 July 2021

[CR72] Naylor-Wardle J, Rowland B, Kunad V (2021). Socioeconomic status and cardiovascular health in the COVID-19 pandemic. Heart.

[CR73] Newalla A, Leonga R, Nazarenoa A, Muscatelloa D, Wooda J, Kimb W (2020). Delay-adjusted age- and sex-specific case fatality rates for COVID-19 in South Korea: evolution in the estimated risk of mortality throughout the epidemic. Int J Infect Dis.

[CR74] Niekerk J, Bakka H, Rue H (2021). Competing risks joint models using R-INLA. Stat Model.

[CR75] Nordström P, Ballin M, Nordström A (2021). Association between risk of COVID-19 infection in nonimmune individuals and COVID-19 immunity in their family members. JAMA Intern Med.

[CR76] Olmo J, Sanso-Navarro M (2021). Modeling the spread of COVID-19 in New York City. Pap Reg Sci.

[CR77] Onder G, Rezza G, Brusaferro S (2020). Case-fatality rate and characteristics of patients dying in relation to COVID-19 in Italy. JAMA.

[CR78] Osei FB, Stein A, Ofosu A (2019). Poisson-Gamma mixture spatially varying coefficient modeling of small–region intestinal parasites infection. Int J Environ Res.

[CR79] Owen B, (2021) Sweden could have avoided four in 10 Covid deaths with early lockdown Availabe at: https://www.thenationalnews.com/world/europe/sweden-could-have-avoided-four-in-10-covid-deaths-with-early-lockdown-1.1221427. Accessed 12 May 2021

[CR80] Pal R, Pal R (2017). Validation methodologies. Predictive modeling of drug sensitivity.

[CR81] Pashakhanlou A (2021). Sweden's coronavirus strategy: the public health agency and the sites of controversy. World Med Health Policy..

[CR82] Paterlini M (2020). Covid-19: Sweden considers tougher restrictions as ICU beds near capacity. BMJ.

[CR83] Payne E, Hardin J, Egede L, Ramakrishnan V, Selassie A, Gebregziabher M (2017). Approaches for dealing with various sources of overdispersion in modeling count data: Scale adjustment versus modeling. Stat Methods Med Res.

[CR84] PHAS (2021) COVID-19. Retrieved at: https://www.cdc.gov/coronavirus/2019-ncov/symptoms-testing/testing.html. PHAS. Accessed 21 May 2021

[CR85] Poirier C, Luo W, Majumder M, Liu D, Mandl K, Mooring T, Santillana M (2020). The role of environmental factors on transmission rates of the COVID-19 outbreak: an initial assessment in two spatial scales. Sci Rep.

[CR86] Riebler A, Held L, Rue H (2011) Modelling seasonal patterns in longitudinal profiles with correlated circular random walks. In: 26th international workshop on statistical modelling, Valencia, 11 July 2011–15 July 2011, pp 503–508

[CR87] Roden L (2020) This is a massive upscaling: Stockholm's Karolinska hospital on increasing Coronavirus testing capacity. Retrieved at: https://sverigesradio.se/artikel/7458128. Accessed 30 June 2021

[CR88] Rouamba T, Samadoulougou S, Kirakoya-Samadoulougou F (2020). Addressing challenges in routine health data reporting in Burkina Faso through Bayesian spatiotemporal prediction of weekly clinical malaria incidence. Sci Rep.

[CR89] Rue H, Held L (2005). Gaussian Markov random fields: theory and applications.

[CR90] Rue H, Martino S, Chopin N (2009). Approximate Bayesian inference for latent Gaussian models by using integrated nested Laplace approximations. J R Stat Soc Ser B Stat Methodol.

[CR91] Rue H, Riebler A, Sørbye S, Illian J, Simpson D, Lindgren F (2017). Bayesian computing with INLA: a review. Annu Rev Stat Appl.

[CR92] Sahu S, Böhning D (2021). Bayesian spatio-temporal joint disease mapping of Covid-19 cases and deaths in local authorities of England. Spat Stat.

[CR93] Sammut C, Webb G, Sammut C, Webb G (2010). Leave-one-out cross-validation BT. Encyclopedia of Machine Learning.

[CR94] Santa F, Henriques R, Torres-Sospedra J, Pebesma E (2019). A statistical approach for studying the spatio-temporal distribution of geolocated tweets in urban environments. Sustainability.

[CR95] Schrödle B, Held L (2011). Spatio-temporal disease mapping using INLA. Environmetrics.

[CR96] Scobie H, Johnson A, Suthar A, PharmD Severson R, Alden N (2021). Monitoring Incidence of COVID-19 Cases Hospitalizations and Deaths by Vaccination Status - 13 US Jurisdictions April 4–July 17 2021. Morb Mortal Wkly Rep.

[CR97] Sellon D, Long M (2014). Equine infectious diseases.

[CR98] Serhiyenko V, Mamun S, Ivan J, Ravishanker N (2016). Fast Bayesian inference for modeling multivariate crash counts. Anal Methods Accid Res.

[CR99] Sidén P, Lindgren F, Bolin D, Villani M (2018). Efficient covariance approximations for large sparse precision matrices. J Comput Graph Stat.

[CR100] SOU (2021a) Sverige under pandemin Volym 1 Smittspridning och smittskydd. Elanders Sverige AB, Stockholm

[CR101] SOU (2021b) Sverige under pandemin Volym 2 Sjukvård och folkhälsa. Elanders Sverige AB, Stockholm

[CR102] Southall E, Tildesley M, Dyson L (2020). Prospects for detecting early warning signals in discrete event sequence data: application to epidemiological incidence data. PLoS Comput Biol.

[CR103] Spiegelhalter D, Best N, Carlin B, Av L (2002). Bayesian measures of model complexity and fit. J R Stat Soc Series B Stat Methodol.

[CR104] Strålin K, Walther S, Holm J, Wahlström E, Bark A, Heurgren M, Lindén T, Hanberger H (2020) Nation-wide study of covid-19 care in Swedish hospitals – 81 % discharged alive. Socialstyrelse, Sweden. Available at: https://www.socialstyrelsen.se/globalassets/1-globalt/covid-19-statistik/engelska-sidan/article-covid19-care-in-swedish-hospitals.pdf. Accessed 12 October 2021

[CR105] Tierney L, Kadane J (1986). Accurate approximations for posterior moments and marginal densities. J Am Stat Assoc.

[CR106] Varghese G, John R, Manesh A, Karthik R, Abraham O (2020). Clinical management of COVID-19. Indian J Med Res.

[CR107] Vicente G, Goicoa T, Ugarte M (2020). Bayesian inference in multivariate spatio-temporal areal models using INLA: analysis of gender-based violence in small areas. Stoch Environ Res Risk Assess.

[CR108] Wakefield J (2007). Disease mapping and spatial regression with count data. Biostatistics.

[CR109] Waller L, Gotway C (2004). applied spatial statistics for public health data.

[CR110] Waller LA, Carlin BP (2010). Disease mapping. Chapman Hall CRC Handb Modern Stat Methods.

[CR111] Wang X, Yue YR, Faraway J (2018). Bayesian regression modeling with INLA.

[CR112] Wang L, Xu C, Wang J, Qiao J, Yan M, Zhu Q (2021). Spatiotemporal heterogeneity and its determinants of COVID-19 transmission in typical labor export provinces of China. BMC Infect Dis.

[CR113] Watanabe S (2010). Asymptotic equivalence of bayes cross validation and widely applicable information criterion in singular learning theory. J Mach Learn Res.

[CR114] Wen R, Torkkola K, Narayanaswamy B, and Madeka D (2018) A multi-horizon quantile recurrent forecaster. arXiv:171111053v2 1–9

[CR115] WHO (2020) Coronavirus disease (COVID-19) Herd immunity lockdowns and COVID-19. WHO. Retrieved at: https://www.whoint/news-room/q-a-detail/herd-immunity-lockdowns-and-covid-19. Accessed 30 October 2021

[CR116] WHO (2021) COVID-19 clinical management: living guidance. WHO. Retrive at: https://www.who.int/publications/i/item/WHO-2019-nCoV-clinical-2021-2. License: CC BY-NC-SA 30 IGO. Accessed 30 October 2021

[CR117] Winkelmann J, Panteli D, Berger E, Busse R (2022). Have we learnt the right lessons? Intensive care capacities during the COVID-19 pandemic in Europe. Eurohealth.

[CR118] Wu H, Wu C, Lu Q, Ding Z, Xue M, Lin J (2021). Spatiotemporal analysis and the characteristics of the case transmission network of 2019 novel coronavirus disease (COVID-19) in Zhejiang Province China. PLoS ONE.

[CR119] Yin P, Mu L, Madden M, Vena J (2014). Hierarchical Bayesian modeling of spatio-temporal patterns of lung cancer incidence risk in Georgia USA: 2000–2007. J Geogr Syst.

