# Supplementary material for: A joint Bayesian spatiotemporal risk prediction model of COVID-19 incidence, IC admission, and death with application to Sweden
Source: Ann Reg Sci. 2022 Nov 28:1–34. Online ahead of print. doi: 10.1007/s00168-022-01191-1 (PMC9707215; doi:10.1007/s00168-022-01191-1)
Supplement: Supplementary file 1 — Supplementary file1 (DOCX 1217 kb) [file 168_2022_1191_MOESM1_ESM.docx]

**Online Resource 1**

**
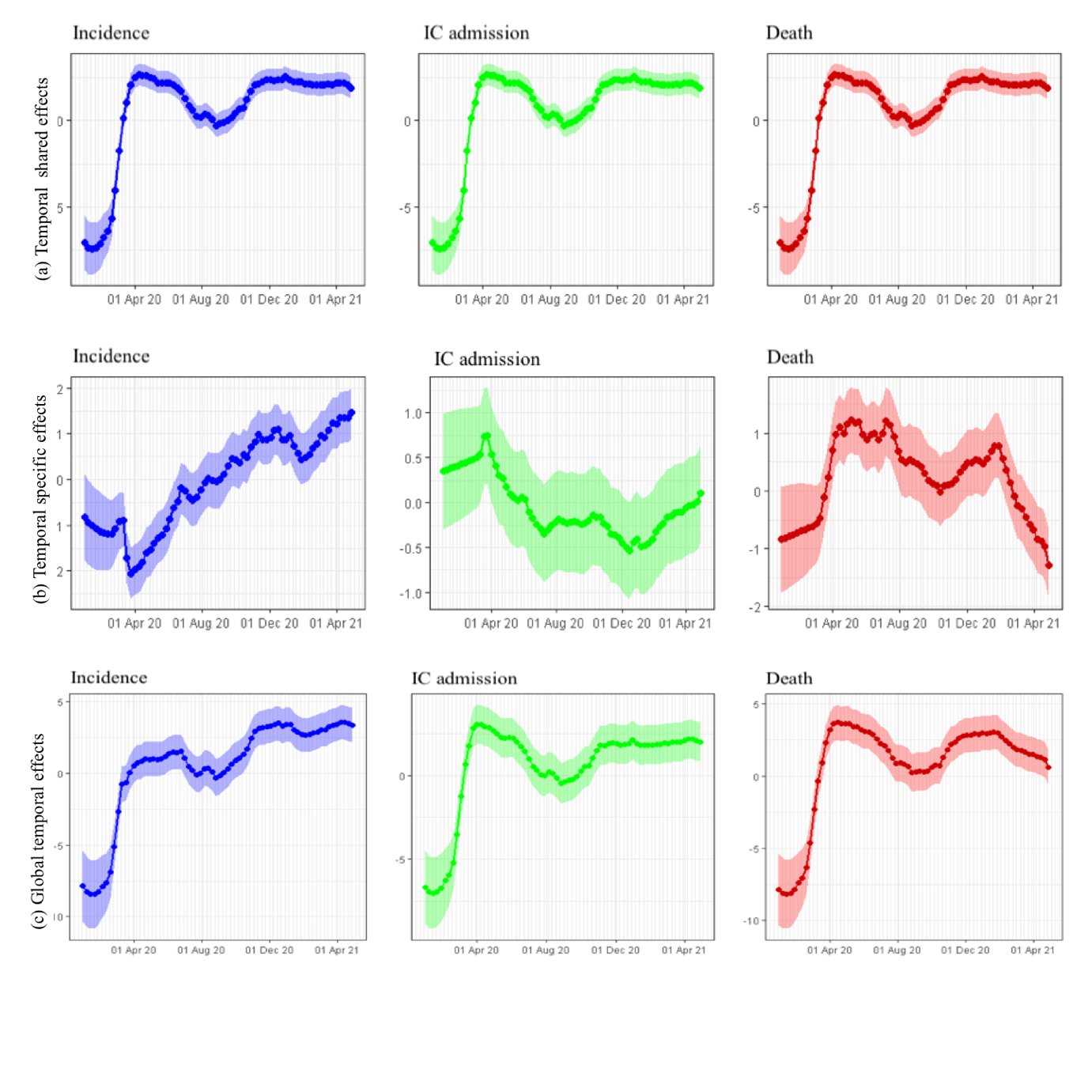
**

**Fig. A1** Temporal effects for incidence, IC admission and death. (a) Temporal shared effect $(\tilde{\phi}_{t}^{y}=\tilde{\phi}_{t}^{o}=\tilde{\phi}_{t}^{z}=\tilde{\phi}_{t})$, (b) Temporal specific effect of incidence ($\ddot{\phi}_{t}^{y})$, IC admission ($\ddot{\phi}_{t}^{o})$, and death ($\ddot{\phi}_{t}^{z}$), and (c) Global temporal effects of incidence ($\phi_{t}^{y})$, IC admission ($\phi_{t}^{o})$, and death ($\phi_{t}^{z}$)

**
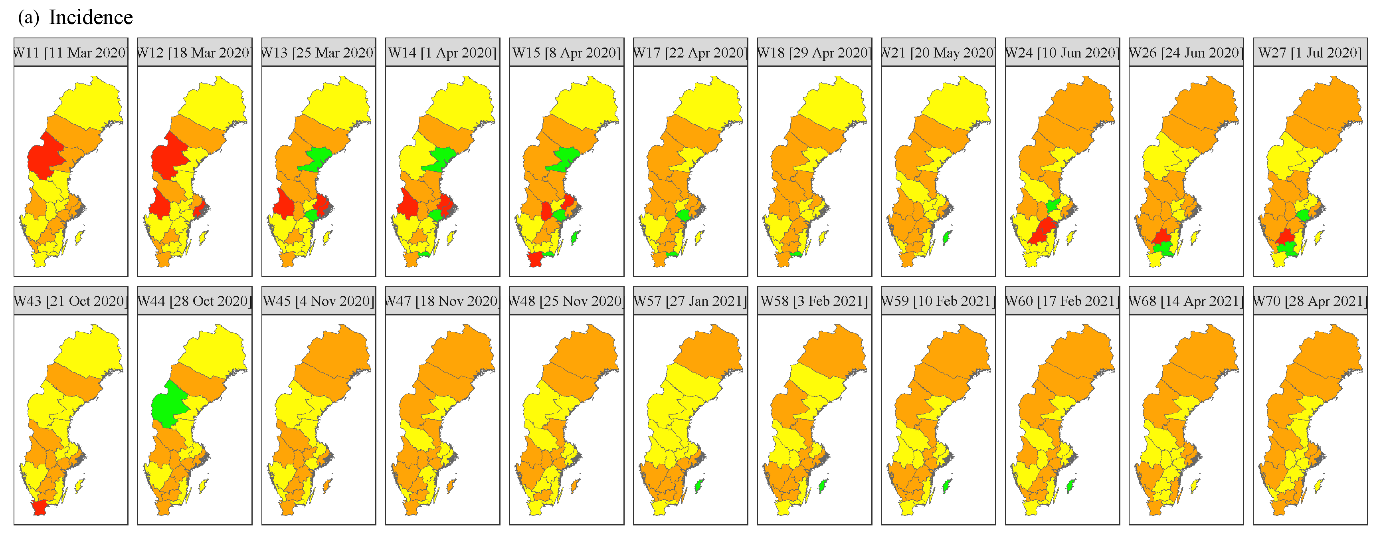

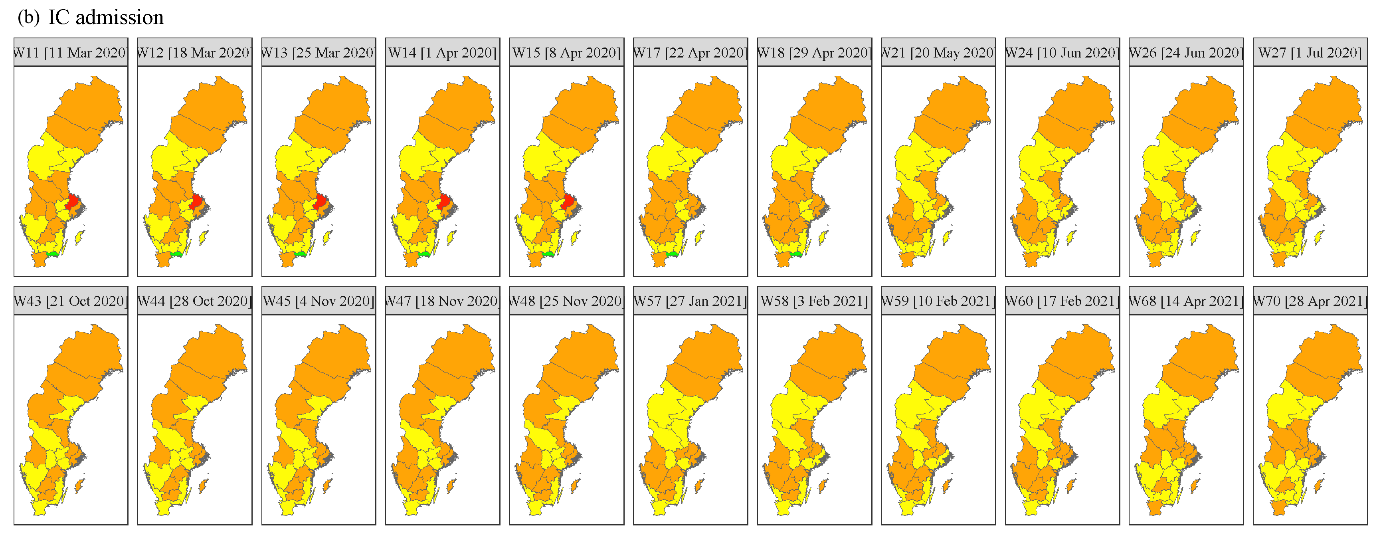

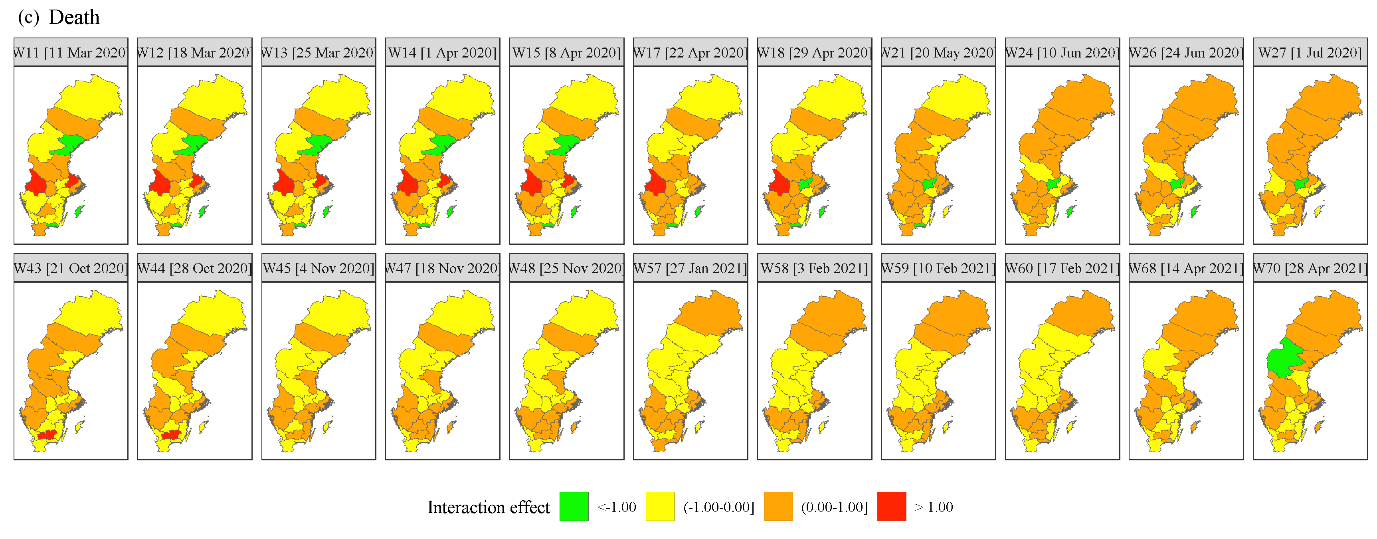
**

**Fig. A2** Estimated interaction effect of (a) incidence, (b) IC admission, and (c) death for selected weeks for W11-15 (11 Mar 2020-8 Apr 2020), W17 (22 Apr 2020), W18 (29 Apr 2020), W21 (20 May 2020), W24 (10 Jun 2020), W26 (24 Jun 2020), W27 (1 Jul 2020), W43 (21 Oct 2020), W44 (28 Oct 2020), W45 (4 Nov 2020), W47(18 Nov 2020), W48 (25 Nov 2020), W57 (27 Jan 2021), W58 (3 Feb 2021), W59 (10 Feb 2021), W60 (17 Feb 2021), W68 (14 Apr 2021), W70 (28 Apr 2021)


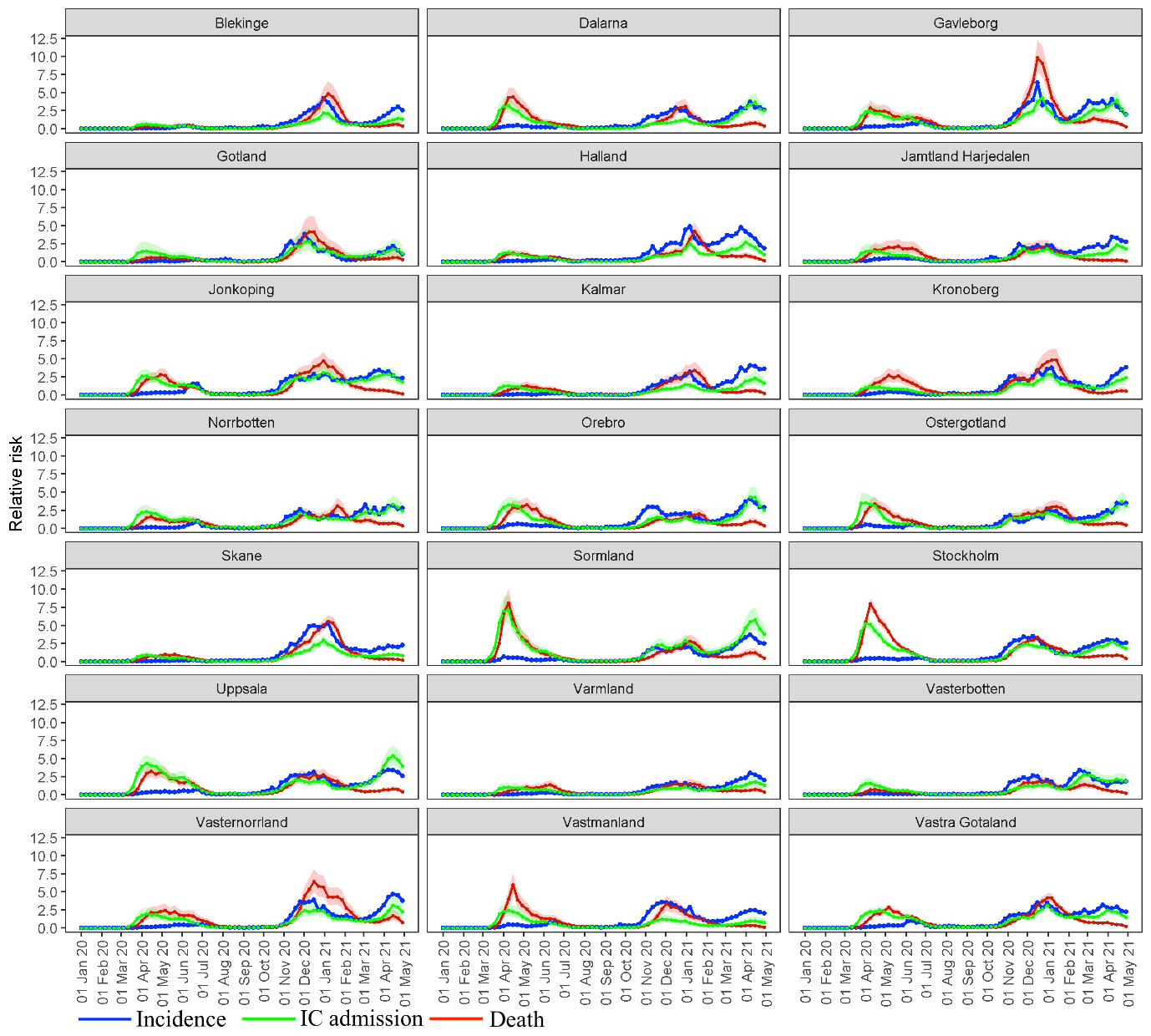


**Fig. A3** Relative risk estimates of incidence, IC admission, and death for period 1 January 2020 to 4 May 2021
